# Group-level workplace interventions to improve mental health in low control, high-demand office-based jobs. A scoping review

**DOI:** 10.1093/annweh/wxae012

**Published:** 2024-03-15

**Authors:** Evangelia Demou, Carolyn Blake, Charisse Tan Llorin, Maria Guadalupe Salanga, Niño Jose Mateo, Ruth Lewis, Kirstin R Mitchell

**Affiliations:** MRC/CSO Social and Public Health Sciences Unit, School of Health and Wellbeing, University of Glasgow, Clarice Pears Building, 90 Byres Road, Glasgow G12 8TB, United Kingdom; MRC/CSO Social and Public Health Sciences Unit, School of Health and Wellbeing, University of Glasgow, Clarice Pears Building, 90 Byres Road, Glasgow G12 8TB, United Kingdom; Social Development Research Center, De La Salle University, 2401 Taft Avenue, Manila 0922, Philippines; Department of Psychology, De La Salle University, 2401 Taft Avenue, Manila 0922, Philippines; Department of Counseling and Educational Psychology, De La Salle University, 2401 Taft Avenue, Manila 0922, Philippines; MRC/CSO Social and Public Health Sciences Unit, School of Health and Wellbeing, University of Glasgow, Clarice Pears Building, 90 Byres Road, Glasgow G12 8TB, United Kingdom; MRC/CSO Social and Public Health Sciences Unit, School of Health and Wellbeing, University of Glasgow, Clarice Pears Building, 90 Byres Road, Glasgow G12 8TB, United Kingdom

**Keywords:** group-level, intervention, low autonomy, mental health, occupational health, office based, workplace intervention, workplaces

## Abstract

**Objectives:**

Workplace psychosocial risk factors, including low autonomy and high demands, have negative consequences for employee mental health and wellbeing. There is a need to support employees experiencing mental health and well-being problems in these jobs. This scoping review aims to describe group-level workplace interventions and their approaches to improving the mental health and well-being of employees in office-based, low autonomy, and high demands jobs.

**Methods:**

Following PRISMA-ScR guidelines, a search was conducted across 4 databases (Medline, PsycINFO, CINAHL, ASSIA). We explored studies presenting group-level interventions, mode of implementation, facilitators and barriers, and intervention effectiveness. The search was restricted to include office-based, low autonomy, and high-demands jobs. Primary outcome of interest was mental health and secondary outcomes were work-related and other well-being outcomes.

**Results:**

Group-level workplace interventions include an array of organizational, relational, and individual components. Almost all included a training session or workshop for intervention delivery. Several had manuals but theories of change were rare. Most workplace interventions did not use participatory approaches to involve employees in intervention development, implementation and evaluation, and challenges and facilitators were not commonly reported. Key facilitators were shorter intervention duration, flexible delivery modes, and formalized processes (e.g. manuals). A key barrier was the changeable nature of workplace environments. All studies employing behavioural interventions reported significant improvements in mental health outcomes, while no clear pattern of effectiveness was observed for other outcomes or types of interventions employed.

**Conclusions:**

Group-based interventions in low-autonomy office settings can be effective but few studies used participatory approaches or conducted process evaluations limiting our knowledge of the determinants for successful group-based workplace interventions. Involving stakeholders in intervention development, implementation, and evaluation is recommended and can be beneficial for better articulation of the acceptability and barriers and facilitators for delivery and engagement.

**What’s Important About This Paper?**

Workplace interventions can create enabling environments for promoting and improving mental health and group-level workplace interventions in particular can have several advantages. This review identified a ‘menu’ of group-level interventions and intervention components that can be used to improve the mental health of office-based employees in jobs with high strain and low autonomy. This is an occupational group who often report poor mental and physical health, increased stress, and low job satisfaction. The findings can help inform the development of other workplace mental health interventions for similar workplaces and employee groups.

## Introduction

Employment, work, and specific job tasks are known determinants of health and extensive research has demonstrated a clear association between the quality of work and health outcomes (Leka *et al.*, 2010; Marmot *et al.*, 2010; Tinson, 2020). Negative working environments, the organization and structure of work, and work with significant psychosocial risk factors can directly and indirectly lead to physical and mental health problems. These can all also impact performance and productivity (Leka *et al.*, 2010; WHO, 2022). Many work-related risk factors for mental health relate to interactions between the type of work, the organizational and managerial environment, the skills and competencies of employees, and the support available for employees to carry out their work (Belloni *et al.*, 2022; WHO, 2022). Psychosocial hazards and aspects of job content are also important risk factors for mental health and well-being (Leka *et al.*, 2010). These include low value, control, and autonomy of work, the low use of skills, lack of task variety and repetitiveness in work, high and conflicting demands, and insufficient resources (e.g. call centre employees) (Errighi *et al.*, 2016; Zito *et al.*, 2018). Also, risk may be increased in situations where there is a lack of team cohesion or social support (WHO, 2022).

Organizations can take effective actions to promote employee mental health in the workplace (Cancelliere *et al.*, 2011; Odeen, Magnussen, *et al.*, 2013). There is evidence that workplace interventions can be effective and can offer a unique entry point into wide segments of the population not only for mental health-related problems but for other health issues and behaviours as well, including pain, weight loss, and other physical health aspects (Czabala *et al.*, 2011; Demou *et al.*, 2018). Workplace interventions can create enabling environments for promoting and improving mental health, reducing stigma and increasing awareness, and behaviour change but few have been rigorously evaluated (Hesketh *et al.*, 2020).

Evidence suggests that linking workplace interventions to organizational objectives, high-level management support, and having effective communication channels in place is important to establishing sustainable workplace interventions (Goetzel and Ozmlnkowski, 2008; Quintiliani *et al.*, 2008). Organizational-level workplace interventions are thought to produce more sustainable effects on the health of employees rather than interventions targeting individual behaviours (Montano *et al.*, 2014). Interventions that appear to be more effective include those with an environmental/organizational and multi-component structure delivered at worksites, during worktime; and interventions that involved staff and included policy changes (Sorensen *et al.*, 1998; Holdsworth *et al.*, 2000; Hunt *et al.*, 2000; Beresford *et al.*, 2001; Conn *et al.*, 2009; Kahn-Marshall and Gallant, 2012; Kaspin *et al.*, 2013). Interventions that involve opportunities for workers to be involved in the decision-making process of the types of interventions and how these are delivered are suggested to more reliably improve worker well-being (Fox *et al.*, 2022). For mental health, two recent systematic meta-reviews suggest that cognitive–behavioural-based stress management interventions can be effective and have positive effects on employee mental health (Joyce *et al.*, 2016; Proper and van Oostrom, 2019). Psychological interventions (Holman *et al.*, 2010; Holman and Axtell, 2016; Proper and van Oostrom, 2019) and e-mental health (via the internet, mobile phones) cognitive behavioural therapy (CBT) interventions focused on making changes on an individual level are effective and can moderate the effects of stress, and burnout (Phillips *et al.*, 2019). These often use psychoeducation focused on CBT in coping skills and resilience training and may be helpful in dealing with high pressure, low autonomy and harassment from clients (Errighi *et al*., 2016). Mindfulness as a workplace intervention also reduces stress (Shonin *et al.*, 2014), improves work-related strain on work-life balance (Michel *et al.*, 2014), increases resilience at work (Aikens *et al.*, 2014) and can improve a number of other well-being outcomes including burnout, sleep issues and psychological distress (Lu *et al.*, 2021).

Group-level workplace interventions delivered to change structural elements of the organization of work, the workplace environment, working conditions, and employee behaviours are also important (Sorensen *et al.*, 1998; Odeen, Ihlebæk, *et al.*, 2013). Group-level interventions have several advantages as they are usually based on peer support for behaviour change, can be cost-effective, are often the preferred option for healthy lifestyle initiatives and they can complement individually focused wellness initiatives (Sorensen *et al.*, 1998; Odeen, Ihlebæk, *et al.*, 2013; Demou *et al.*, 2018). However, there is a gap in understanding the potential contribution of group-level workplace interventions and whether these can be effective for specific occupational groups and organizational contexts (Hesketh *et al.*, 2020; Fox *et al.*, 2022).

While previous literature has demonstrated that workplace interventions can improve mental health and well-being, the diversity in workplaces, interventions, and outcomes prevents robust conclusions from being drawn (Hesketh *et al.*, 2020) on the transferability and applicability of these to specific types of workplaces. To provide a more nuanced understanding of a particular intervention approach and context, this review asks: what group-level workplace interventions work best for office-based employees in jobs with high strain and low autonomy? and what are the barriers and facilitators for successful intervention development, implementation, and evaluation? The questions are posed in the context of a larger project on the mental health of call-centre employees which sought to co-design an intervention with employees and employers (Mitchell, 2021). Call centre employees are an occupational group who often report poor mental and physical health, increased stress, and low job satisfaction. We synthesize and describe the range and scope of studies presenting group-level interventions, their mode of implementation, the intervention components, facilitators, and barriers in implementation, and the extent to which these interventions are effective.

## Methods

The scoping review followed the PRISMA-ScR guidelines (Tricco *et al.*, 2018) (see PRISMA-ScR Checklist).

### Inclusion criteria

We included any studies that described group-level interventions delivered in workplace settings, specifically targeting employees in desk/office-based work and in occupations with low autonomy (i.e. low job control (Karasek, 1985)) and high stress. As workplace interventions can influence employee wellbeing across many domains, the interventions included had to explore and report a mental health (e.g. anxiety, depression) and/or wellbeing (e.g. mindfulness, sleep, social support) outcome of interest, but mental health did not have to be the primary focus of the intervention. For instance, interventions with primary goals to increase productivity, improve stress management, well-being, fitness, and weight management could all be included if they also examined impacts on mental health. Our primary outcome of interest was a mental health outcome (e.g. depression, anxiety, stress, burnout, mood, fatigue, and emotional exhaustion); and secondary outcomes of interest included work-related outcomes (e.g. including productivity, morale, sickness absence, need-for-recovery (NfR), workability, job performance, and job strain); and other outcomes of interest (e.g. mindfulness, wellbeing/physical health, self-compassion, energy levels, sleep, and social support). As previous studies suggest- and to be consistent with our intervention design project (Mitchell, 2021)— that including employee engagement in the development, implementation, and evaluation of the intervention is beneficial (Fox *et al.*, 2022), we explored whether the interventions used a participatory approach in intervention development, implementation and/or evaluation. Studies were included if they were published in the scientific peer-reviewed literature, from 2000 to present to capture current working conditions, in English or French. There was no restriction on study design. The target population was working-age adults in high- and middle-income countries, as our interest was in low-autonomy jobs that are desk/office-based such as call centre work. Outcomes of interest were mental health-related outcomes, including anxiety, depression, stress, burnout, and well-being.

### Exclusion criteria

Interventions delivered to the self-employed or employees working in small to medium enterprises (SMEs) were not included. Studies were excluded if interventions were delivered in low-income countries, due to stark differences in labour market, and employment conditions (Kapsos and Bourmpoula, 2013; Lam and Elsayed, 2021) or if they were delivered in a work setting that the research team agreed was not likely to involve work that was similar/representative of the desk/office-based jobs. Such work and workplaces included manual jobs (e.g. mining industry), healthcare and community health settings, and sex work. Additionally, studies detailing interventions that are delivered to individuals or interventions targeting patient groups (even if they were workers) were excluded (Hesketh *et al.*, 2020).

### Search strategy

The author team met several times to discuss the search strategy and terms to be used, following the PICOC framework (Mengist *et al.*, 2019). We further consulted with our in-house information scientist, who tested and developed the full search string and carried out the searches. Our final search strategy included search terms for (i) population based on occupation (e.g. white collar, workplace, employee, worker, call centres/contact centres, and low autonomy work); (ii) intervention (e.g. promotion, intervention, evaluation, prevention, program, and participatory approach); and (iii) outcome of interest (e.g. mental health, mental illness, mental disorder, depression, wellbeing/well-being, anxiety, stress, psychological, burnout, control, strain, and demand); and (iv) context (high and middle-income countries). The search strategy syntax was adapted to the specific requirements of each different database used and the exact search string is presented in Supplementary Table S1.

**Figure 1.**
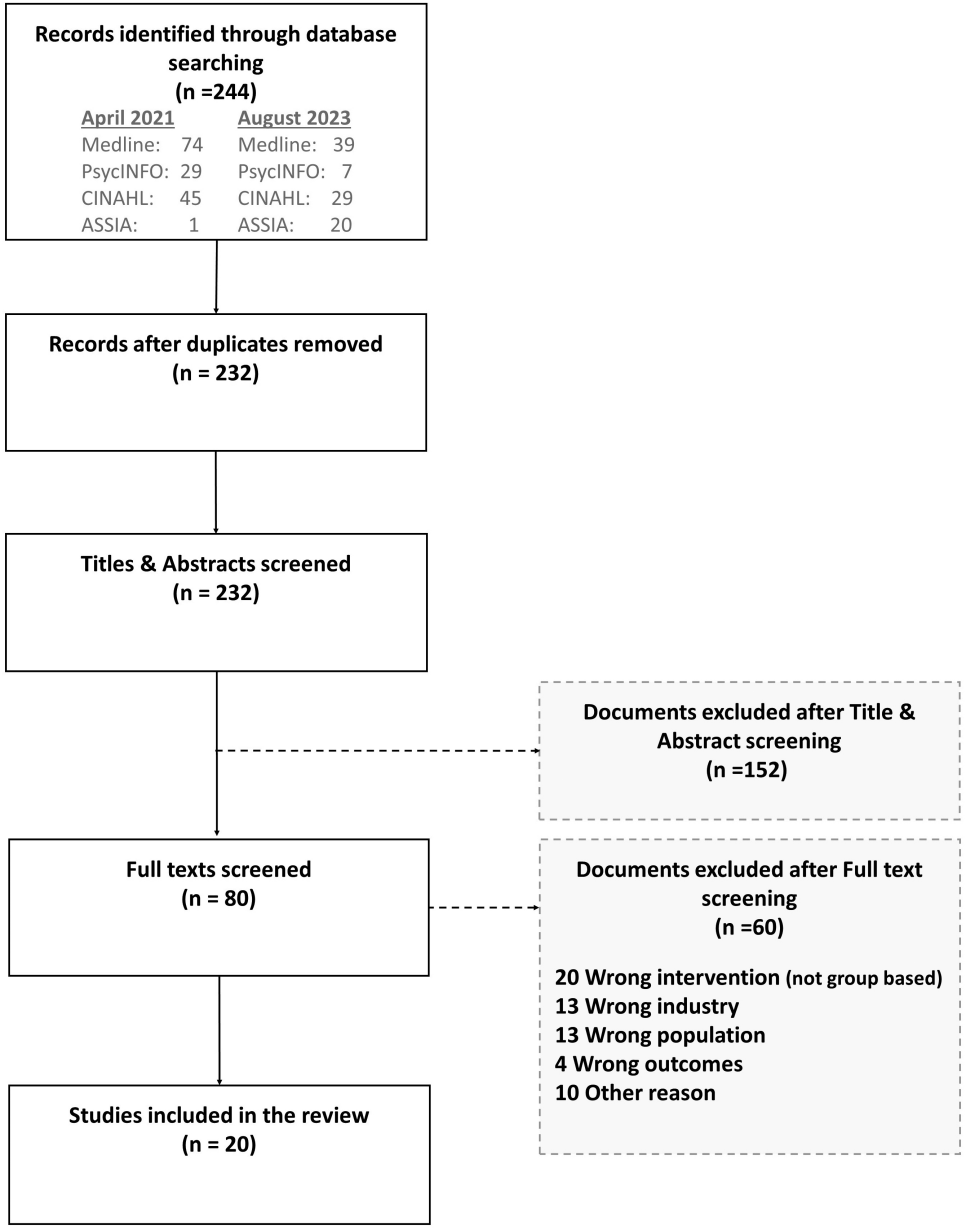
Flow chart of the selection process of included studies.

To be as inclusive as possible, we consulted databases across the medical, public health, and social science disciplines: Medline, PsycINFO, CINAHL, and ASSIA. Most authors participated in screening the titles using COVIDENCE (Covidence, 2022), abstracts, and full papers using the inclusion criteria defined in the previous paragraphs. Each title obtained from the electronic search (Fig. 1) was independently assessed by two reviewers, and where they did not agree, a third reviewer was consulted to reach a consensus. After title screening, abstracts were independently screened for eligibility by two reviewers and any discrepancies were resolved by discussion. During full-text screening, reasons for exclusion were noted and discussed by the reviewers.

### Data extraction

To develop the final data extraction table, members of the research team used a pilot version to independently extract data from a sub-sample of the included papers and refine the data extraction fields. The final version contained fields to extract information pertaining to the article details (e.g. first author and publication year); country; study design (including any participatory elements); workplace setting; study participants/sample size, intervention aim; and intervention description (including components), intervention outcomes, effectiveness, and/or cost-effectiveness, as well as on challenges and facilitators.

### Analysis

To identify themes and key intervention components from the reviewed studies, a narrative synthesis approach was undertaken using three distinct steps: collating, summarizing, and reporting the results (Levac *et al.*, 2010). The information was collated in tables, and the main findings were summarized and reported by outcome of interest. This involved an iterative process, examining the evidence for intervention components that may have influenced the outcomes.

### Evidence synthesis (effectiveness)

To assess the effectiveness of group-level workplace interventions, we performed an evidence synthesis based on significance or non-significance in relation to our primary outcome of interest: mental health outcomes (e.g. depression, anxiety, stress, burnout, mood, fatigue, and emotional exhaustion); and our secondary outcomes of interest: work-related outcomes (e.g. including productivity, morale, sickness absence, need-for–recovery (NfR), workability, job performance, and job strain); and other outcomes of interest (e.g. mindfulness, wellbeing/physical health, self-compassion, energy levels, sleep, and social support). A scoring system adapted from previous reviews (Hoogendoorn *et al.*, 2000) was implemented where each study was given equal weight, and if the study reported significant improvement in outcome it was given a score of one (1) for the outcome of interest; a score of zero (0) if there was a non-significant change or inconsistent results reported and a score of negative one (−1) if the intervention had a significant negative effect. Scores per outcome were added and represented as a percentage of the maximum possible score. The criteria used for the evidence synthesis were: “sufficient evidence”—if score was 50% or higher; “moderate evidence”—if the score was between 25% and 50%; “insufficient evidence”—for scores less than 25%.

## Results

### Characteristics of included studies

Our search identified 244 studies (first search was completed on 14th April 2021; the search updated on 14th August 2023). After title screening, 80 full texts were independently screened for eligibility and 60 papers were excluded at this stage. In total 20 studies were included in our scoping review (Fig. 1). The main reasons for exclusion included: (i) wrong intervention type (e.g. the intervention was not a group workplace intervention; *n* = 19); (ii) studies covered workers in occupations that were not office-based and were not considered to have jobs with low autonomy; *n* = 13, (iii) the population covered were not workers (e.g. patient groups; *n* = 8); (iv) there were no mental health outcomes of interest; *n* = 4; and (v) other reasons; *n* = 10 (e.g. not peer-reviewed studies; theses).

**Figure 2.**
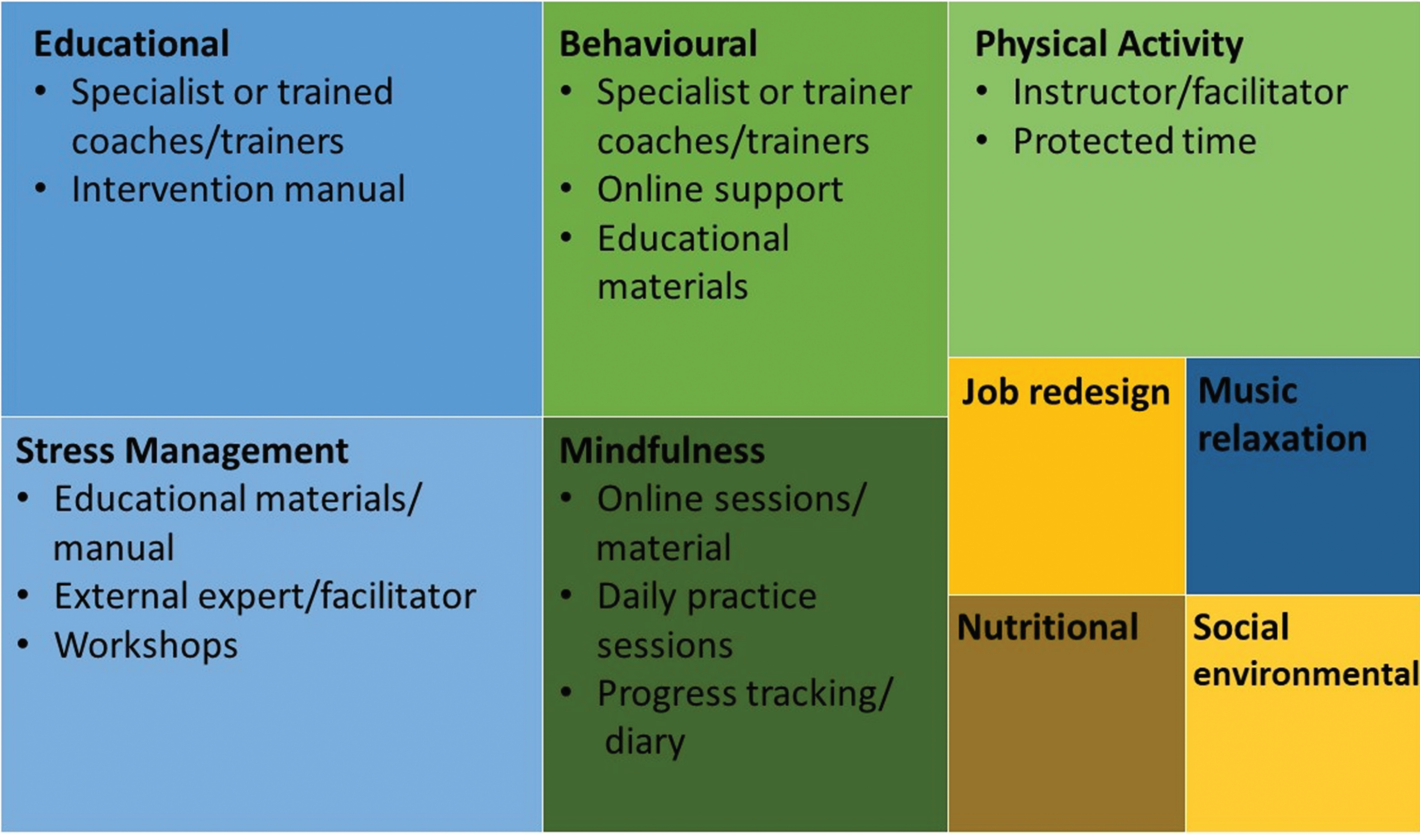
Group-level intervention types and common intervention components (box size scaled to proportion of identified interventions).

Overall, the included studies covered 20 different interventions (Table 1 and Supplementary Table S1, Figure 2) (Munz *et al.*, 2001; Workman and Bommer, 2004; Takao *et al.*, 2006; Mills *et al.*, 2007; Smith, 2008; Hasson *et al.*, 2010; Kojima *et al.*, 2010; Ahola *et al.*, 2012; Aikens *et al.*, 2014; Dollard and Gordon, 2014; Agarwal *et al.*, 2015; Grégoire and Lachance, 2015; Formanoy *et al.*, 2016; Saelid *et al.*, 2016; Arredondo *et al.*, 2017; Lloyd *et al.*, 2017; Michishita *et al.*, 2017; Das *et al.*, 2019, 2020; Saavedra *et al.*, 2021). All identified studies were conducted in high-income settings. The studies cover 4 continents, with 8 from Europe (Mills *et al.*, 2007; Hasson *et al.*, 2010; Ahola *et al.*, 2012; Formanoy *et al.*, 2016; Saelid *et al.*, 2016; Arredondo *et al.*, 2017; Lloyd *et al.*, 2017; Saavedra *et al.*, 2021), 7 from North America (6 from the USA (Munz *et al.*, 2001; Workman and Bommer, 2004; Aikens *et al.*, 2014; Agarwal *et al.*, 2015; Das *et al.*, 2019, 2020) and 1 from Canada (Grégoire and Lachance, 2015)), 3 from Japan (Takao *et al.*, 2006; Kojima *et al.*, 2010; Michishita *et al.*, 2017) and 2 from Australia (Smith, 2008; Dollard and Gordon, 2014). More than half of the studies were randomized controlled studies (Takao *et al.*, 2006; Smith, 2008; Hasson *et al.*, 2010; Ahola *et al.*, 2012; Aikens *et al.*, 2014; Agarwal *et al.*, 2015; Formanoy *et al.*, 2016; Saelid *et al.*, 2016; Arredondo *et al.*, 2017; Lloyd *et al.*, 2017; Michishita *et al.*, 2017; Das *et al.*, 2019; Saavedra *et al.*, 2021), 4 were pre–post-intervention studies (Munz *et al.*, 2001; Workman and Bommer, 2004; Grégoire and Lachance, 2015; Das *et al.*, 2020) and 3 studies had a quasi-experimental design (Mills *et al.*, 2007; Dollard and Gordon, 2014; Saavedra *et al.*, 2021). The studies covered a wide range of workplaces with office-based work and tasks that were deemed to be of low job control and high demands desk/office-based jobs, from corporate settings such as insurance, information technology, banking, and financial sectors, government and public bodies, universities, not for profit workplaces, clinical research centre, call centres, and manufacturing. The studies covered 6017 employees overall, with the smallest study covering 47 participants (Saavedra *et al.*, 2021) and the largest 1679 participants (Mills *et al.*, 2007).

**Table 1.** Descriptive table of included studies

Study ID—country	Setting	Target outcomes (measurement tool)	Type of intervention	Participatory approach used
(Agarwal *et al.*, 2015)—USA	10 corporate sites of an insurance company	Primary: depression; anxiety Secondary: work productivity	Nutritional intervention	Not reported
(Ahola *et al.*, 2012)—Finland	17 organizations (9 city administration; 5 government; 3 private companies)	Primary: depression Secondary: job strain; depressive symptoms	Skills training intervention Skills training used active learning methods	Implementation (trainers)
(Aikens *et al.*, 2014)—USA	Chemical industry	Primary: stress; mindfulness; wellbeing	Mindfulness intervention	Implementation
(Arredondo *et al.*, 2017)—Spain	Private international clinical research company	Primary: stress Secondary: mindfulness; heart rate variability; self-compassion; de-centring; burnout	Mindfulness intervention	Not reported
(Das *et al.*, 2019)- USA	12 Broad range of worksites (5 universities; 5 for profit companies; 2 non-profit)	Primary: Employee vitality (energy) Secondary: quality of life; purpose in life; sleep; mood; depression; body mass index	Behavioural intervention	Implementation
(Das *et al.*, 2020)- USA	Broad range of worksites (e.g. 5 universities, 5 for-profit and 2 non-profit organizations)	Primary: vitality; PiL Secondary: sleep, mood, depression, BMI	Behavioural intervention	Not reported
(Dollard and Gordon, 2014)- Australia	Public sector organization	Primary: organizational and job design factors Secondary: stress; morale; sickness absence	Stress management intervention	Intervention design
(Formanoy *et al.*, 2016)- The Netherlands	Financial service provider	Primary: need for recovery (NFR after work scale)	Social environmental and physical activity intervention	Not reported
(Grégoire and Lachance, 2015)- Canada	Call centre in a financial service provider	Primary: mindfulness; stress; anxiety; depression; fatigue; negative affects	Mindfulness intervention	Not reported
(Hasson *et al.*, 2010)- Sweden	Information technology	Primary: stress	1.Stress management intervention 2.Cognitive ergonomics intervention	1.Not reported 2. Intervention design; implementation; evaluation; monitoring
(Kojima *et al.*, 2010)- Japan	Office workers of a metal manufacturing industry	Primary: depression; self-esteem Secondary: an understanding of stress control skills;will to apply these stress control skills	Cognitive behavioural therapy training intervention	Not reported
(Lloyd *et al.*, 2017)- UK	Government departments (customer facing roles)	Primary: psychological strain; emotional exhaustion; depersonalization	CBT-focused stress management intervention	Not reported
(Michishita *et al.*, 2017)- Japan	White-collar	Primary: personal relationships; profile of mood states; physical activity; physical health; work ability	‘Active rest’ intervention (physical activity)	Not reported
(Mills *et al.*, 2007)- UK	Office based employees of a multinational manufacturer of food, home care, and personal care products	Primary: count of health risk factors; sickness absence; work performance Secondary: return on investment due to health risk change; intervention impact on individual health risk factors	Health promotion intervention	Not reported
(Munz *et al.*, 2001)- USA	Telecommunications company in 4 different cities	Primary: emotional wellbeing; productivity; sickness absence Secondary: job independence	Worksite stress management program	Not reported
(Saavedra *et al.*, 2021)- Iceland	Sedentary office work setting	Primary: body composition; cardiorespiratory fitness; lipid profile; blood pressure; mental health	Physical activity interventions: circuit training; brisk walk	Not reported
(Saelid *et al.*, 2016)- Norway	Public organizations	Primary: burnout, depressive symptoms; self-efficacy; quality of life; self-esteem; social support; negative life events; sickness absence	Coping with strain intervention (educational)	Not reported
(Smith, 2008)- Australia	Call centre—customer facing employees	Primary: anxiety	Music relaxation intervention	Not reported
(Takao *et al.*, 2006)- Japan	Japan Sake brewery	Primary: psychological distress; job performance	Job stress education intervention delivered to supervisors	Not reported
(Workman and Bommer, 2004)- USA	Call centre of an international computer company	Primary: job strain; job attitudes	Job redesign—comparison of 3 types of intervention	Not reported

The included studies had to target or explore a mental health outcome of interest, but the primary focus of the intervention did not have to be mental health-focused. Therefore, a broad range of interventions are included in the review (Fig. 2). The most common intervention type focussed on stress management (Munz *et al.*, 2001; Hasson *et al.*, 2010; Dollard and Gordon, 2014; Lloyd *et al.*, 2017). Physical activity (Formanoy *et al.*, 2016; Michishita *et al.*, 2017; Saavedra *et al.*, 2021) and behavioural and interventions using cognitive behavioural techniques (CBT) (Kojima *et al.*, 2010; Lloyd *et al.*, 2017; Das *et al.*, 2019, 2020) were used in 3 intervention designs; while mindfulness (Aikens *et al.*, 2014; Arredondo *et al.*, 2017) and educational (Takao *et al.*, 2006; Saelid *et al.*, 2016) interventions were used in 2 studies each. The remaining types of interventions were only used in one study each and included nutritional (Agarwal *et al.*, 2015), health promotion (Mills *et al.*, 2007), music relaxation (Smith, 2008), and a job redesign (Workman and Bommer, 2004) intervention.

### Intervention delivery

Half of the interventions were solely group-level (Workman and Bommer, 2004; Takao *et al.*, 2006; Smith, 2008; Ahola *et al.*, 2012; Dollard and Gordon, 2014; Saelid *et al.*, 2016; Michishita *et al.*, 2017; Das *et al.*, 2019, 2020; Saavedra *et al.*, 2021), and the other half were mixed interventions including both group-level and individual level components (Munz *et al.*, 2001; Mills *et al.*, 2007; Hasson *et al.*, 2010; Kojima *et al.*, 2010; Aikens *et al.*, 2014; Agarwal *et al.*, 2015; Grégoire and Lachance, 2015; Formanoy *et al.*, 2016; Arredondo *et al.*, 2017; Lloyd *et al.*, 2017) (Table 1 and Supplementary Table S1). The interventions studied were delivered in the workplace (Munz *et al.*, 2001; Workman and Bommer, 2004; Takao *et al.*, 2006; Mills *et al.*, 2007; Smith, 2008; Hasson *et al.*, 2010; Ahola *et al.*, 2012; Dollard and Gordon, 2014; Agarwal *et al.*, 2015; Grégoire and Lachance, 2015; Formanoy *et al.*, 2016; Arredondo *et al.*, 2017; Michishita *et al.*, 2017; Saavedra *et al.*, 2021) or both in the workplace and in non-workplace setting such as online (Kojima *et al.*, 2010; Aikens *et al.*, 2014; Saelid *et al.*, 2016; Lloyd *et al.*, 2017; Das *et al.*, 2020). All interventions but one (Hasson *et al.*, 2010) had components that were delivered in person, such as training sessions and workshops, and 8 of these had an additional online component to supplement the intervention (Mills *et al.*, 2007; Hasson *et al.*, 2010; Kojima *et al.*, 2010; Aikens *et al.*, 2014; Agarwal *et al.*, 2015; Formanoy *et al.*, 2016; Das *et al.*, 2019, 2020). Duration varied considerably with one intervention lasting just 15 min (Smith, 2008), another consisting of 15 min sessions over 5 weeks (Grégoire and Lachance, 2015), to others being delivered over several weeks or months (Hasson *et al.*, 2010; Kojima *et al.*, 2010; Ahola *et al.*, 2012; Aikens *et al.*, 2014; Dollard and Gordon, 2014; Agarwal *et al.*, 2015; Saelid *et al.*, 2016; Arredondo *et al.*, 2017; Lloyd *et al.*, 2017; Michishita *et al.*, 2017; Saavedra *et al.*, 2021). However, for many interventions there was no information on how long the intervention was delivered for (Munz *et al.*, 2001; Workman and Bommer, 2004; Takao *et al.*, 2006; Mills *et al.*, 2007; Formanoy *et al.*, 2016).

Most workplace interventions did not use participatory approaches to involve employees in intervention development, implementation, and evaluation (Munz *et al.*, 2001; Workman and Bommer, 2004; Takao *et al.*, 2006; Mills *et al.*, 2007; Smith, 2008; Hasson *et al.*, 2010; Kojima *et al.*, 2010; Agarwal *et al.*, 2015; Grégoire and Lachance, 2015; Formanoy *et al.*, 2016; Saelid *et al.*, 2016; Arredondo *et al.*, 2017; Lloyd *et al.*, 2017; Michishita *et al.*, 2017; Das *et al.*, 2019; Saavedra *et al.*, 2021) (Table 1 and Supplementary Table S1). One intervention used a participatory approach in the development phase (Dollard and Gordon, 2014), and three interventions did so in the implementation phase for example with the use of a trainer from their employees (Ahola *et al.*, 2012), team leaders acting as champions for the intervention (Aikens *et al.*, 2014) and having an employee contact person to facilitate implementation, e.g. participant recruitment (Das *et al.*, 2019).

### Intervention components

The components of the interventions implemented in the different workplaces were varied (Table 1 and Supplementary Table S1, Fig. 2). All interventions included multi-components and almost all interventions included a training session or workshop for intervention delivery (Munz *et al.*, 2001; Workman and Bommer, 2004; Takao *et al.*, 2006; Mills *et al.*, 2007; Smith, 2008; Kojima *et al.*, 2010; Ahola *et al.*, 2012; Aikens *et al.*, 2014; Dollard and Gordon, 2014; Agarwal *et al.*, 2015; Grégoire and Lachance, 2015; Formanoy *et al.*, 2016; Arredondo *et al.*, 2017; Lloyd *et al.*, 2017; Michishita *et al.*, 2017; Das *et al.*, 2019, 2020; Saavedra *et al.*, 2021). Several included online sessions that mainly supplemented the in-person components (Mills *et al.*, 2007; Hasson *et al.*, 2010; Kojima *et al.*, 2010; Aikens *et al.*, 2014; Agarwal *et al.*, 2015; Formanoy *et al.*, 2016; Das *et al.*, 2019, 2020) and one intervention included audio sessions (Grégoire and Lachance, 2015). Trained experts, e.g. with a clinical background (Munz *et al.*, 2001; Takao *et al.*, 2006; Kojima *et al.*, 2010; Dollard and Gordon, 2014; Agarwal *et al.*, 2015; Formanoy *et al.*, 2016; Saelid *et al.*, 2016; Michishita *et al.*, 2017; Das *et al.*, 2019, 2020) or members from the academic research teams acting as facilitators (Smith, 2008; Saavedra *et al.*, 2021) were also used in the delivery of the interventions. A number of interventions used manuals (Munz *et al.*, 2001; Takao *et al.*, 2006; Mills *et al.*, 2007; Hasson *et al.*, 2010; Dollard and Gordon, 2014; Agarwal *et al.*, 2015; Lloyd *et al.*, 2017; Das *et al.*, 2020), with one including a manual only for the control group (Ahola *et al.*, 2012). Other manuals or materials included record diaries (Arredondo *et al.*, 2017), an intervention checklist (Kojima *et al.*, 2010) or a manual that was used for home assignments for team leaders (Saelid *et al.*, 2016). Other intervention components that were used to facilitate delivery and enhance participation included having a reminder system in place delivered either by text or email (Mills *et al.*, 2007; Hasson *et al.*, 2010; Aikens *et al.*, 2014), rewards or incentives for participants such as protected time within the work day to take part in the intervention (Grégoire and Lachance, 2015; Lloyd *et al.*, 2017; Saavedra *et al.*, 2021), delivering the intervention on site (Grégoire and Lachance, 2015; Lloyd *et al.*, 2017; Michishita *et al.*, 2017), or providing other rewards for intervention participants (Workman and Bommer, 2004) or for the control group (Agarwal *et al.*, 2015).

Tailored participant feedback was an intervention component used in 5 studies (Munz *et al.*, 2001; Mills *et al.*, 2007; Hasson *et al.*, 2010; Kojima *et al.*, 2010; Aikens *et al.*, 2014) (e.g. provided by email (Aikens *et al.*, 2014)). Only one study provided health feedback on blood samples (Hasson *et al.*, 2010).

Environmental interventions were used in 3 different interventions (Agarwal *et al.*, 2015; Formanoy *et al.*, 2016; Saavedra *et al.*, 2021). These included changes to the provision of foods that were served in the workplace cafeteria (Agarwal *et al.*, 2015), to changes to the workplace environment to create more social interactions (Formanoy *et al.*, 2016) for instance. Two studies introduced and provided equipment for the employees such as exercise balls, standing tables, and footsteps to promote stair use (Formanoy *et al.*, 2016; Saavedra *et al.*, 2021). Other components included the use of objective anthropometric indices to assess impact (Michishita *et al.*, 2017), having a mindfulness retreat for employees (Arredondo *et al.*, 2017), and or restructuring the organization of work and reward systems in place (Workman and Bommer, 2004).

### Challenges and facilitators for workplace interventions

Challenges and facilitators were not commonly reported (Table 2). Several of the intervention studies discussed facilitators, mainly focusing on the implementation of the interventions and participant engagement. Interventions that had a published manual were seen as beneficial for delivery across sectors and even commercially available interventions can be used or adapted for workplaces. For intervention participation and engagement, shorter interventions and those that were flexible in their delivery were regarded as being able to assist in overcoming barriers to employee participation.

**Table 2.** Challenges, facilitators and recommendations for evaluation of group-level mental health interventions

Challenges	Facilitators	Recommendations
Representativeness of participants: issues of small samples sizes; no control groups; self-selection of participants; often participants in workplace intervention studies from large, medium size companies in urban settings	Theory-based interventions and especially if a published manual is available, allow interventions to be reproduced in other workplaces/sectors	More studies are needed on different employee groups and larger populations
Randomization at the workplace level, not at the participants level	Workplace employees involved in the design, implementation and/or evaluation of intervention in combination with other professionals	Evaluations on cost-benefit and cost-effectiveness of workplace interventions
Evaluation often relies on self-report measures/outcomes; however, biomarkers can also have limitations as levels may differ to other factors not assessed	Commercially available interventions can be adapted and used in the workplace	Detailed information about the workplace intervention are warranted to enable the interventions to be applied in other workplaces (sectors, industries) and have wider distribution
Inability to control for confounding/mediating factors	Shorter intervention times may make employees more inclined to participate	More organizational outcomes (e.g. productivity, sickness absence, costs) should be examined
Follow-up not always possible (e.g. avoid burdening participants)	Interventions that have flexible delivery, (e.g. delivered in workplace, home, other), can assist in overcoming barriers for participation	Studies should examine varied durations, intensity, sustainability, the use of only self-directed tools in the workplace
Lack of cost-effectiveness/cost-benefit analysis		Tailoring of interventions to specific populations may be needed
Mixed interventions hard to assess mechanisms of change		The use and advantages/disadvantages of experienced trainers/facilitators to be assessed
Lack of time/ability to engage are barriers for engagement		Intervention sessions/components that are more interactive may improve participation
For online forums/chat rooms cannot assess impact of place and time of participation		Organizations should consider ways to set up the work environment that it is inherently motivating in a more proactive way to deal with workplace issues such as job strain
Frequently face-to-face meetings with qualified specialists are impractical/costly		
Conditions during the intervention out of one’s control (e.g. weather for outdoor intervention; changes in workplace practices/policies)		

The main challenge reported for workplace intervention delivery was the fact that, within workplaces, conditions may change which can further impact intervention engagement and evaluation of effectiveness.

Some of the challenges and barriers in synthesizing the evidence on the effectiveness of workplace interventions included the difficulty to assessing representativeness due to relatively small sample sizes, often there was no control group, participants self-selected into the interventions, and interventions that were evaluated generally came from large companies in urban areas. Other challenges for evaluation that were reported across studies included the lack of cost-benefit and cost-effectiveness analysis, changes within the workplace may impact the intervention, and that most of the interventions involve varied components so it is difficult to assess the mechanisms of change.

### Evidence of effectiveness

Evidence synthesis on the effectiveness of the interventions on the mental health of employees, as well as work-related and other outcomes of interest (e.g. mindfulness, wellbeing/physical health, self-compassion, energy levels, sleep, cognitive strain, self-esteem, personal relationships, social support) was performed on all 20 studies. Table 3 indicates that there is strong evidence for improvements in mental health outcomes, and moderate evidence for improvements in work-related and other outcomes (see also Supplementary Table S3). Nine of the studies reporting mental health outcomes showed positive and significant impacts (Munz *et al.*, 2001; Smith, 2008; Hasson *et al.*, 2010; Kojima *et al.*, 2010; Aikens *et al.*, 2014; Agarwal *et al.*, 2015; Saelid *et al.*, 2016; Das *et al.*, 2019, 2020); and 4 studies showed no significant difference between intervention and control groups (Takao *et al.*, 2006; Ahola *et al.*, 2012; Arredondo *et al.*, 2017; Lloyd *et al.*, 2017). For work-related outcomes, 3 studies reported significant improvements (Mills *et al.*, 2007; Agarwal *et al.*, 2015; Das *et al.*, 2019); 6 studies showed non-significant change (Munz *et al.*, 2001; Workman and Bommer, 2004; Takao *et al.*, 2006; Dollard and Gordon, 2014; Formanoy *et al.*, 2016); and one study demonstrated a significant negative effect in cardiometabolic risk factors (Das *et al.*, 2020). Other outcomes of interest related to health and well-being, including mindfulness, energy levels, self-esteem, and social support had three studies reporting significant improvements (Mills *et al.*, 2007; Smith, 2008; Aikens *et al.*, 2014); 3 studies demonstrating non-significant findings (Das *et al.*, 2019, 2020) (Kojima *et al.*, 2010).

**Table 3.** Evidence synthesis table.

Study	Study design	Type of intervention	Outcomes of interest		
			Mental health outcomes ^a^	Work outcomes ^b^	Other outcomes ^c^
Das *et al.* (2019)	RCT	Behavioural			
Das *et al.* (2020)	Pre/post	Behavioural			
Kojima *et al.* (2010)	RCT	Cognitive behavioural therapy			
Ahola *et al.* (2012)	RCT	Skill training (educational)			
Saelid *et al.* (2016)	RCT	Coping with strain (educational)			
Mills *et al.* (2007)	Pre/Post	Health promotion (educational)			
Takao *et al.* (2006)	RCT	Job stress (educational)			
Workman and Bommer (2004)	Pre/Post	Job redesign			
Aikens et al*.* (2014)	RCT	Mindfulness			
Arrendondo *et al.* (2017)	RCT	Mindfulness			
Grégoire and Lachance. (2015)	Pre/Post	Mindfulness			
Smith (2008)	RCT	Music relaxation			
Agarwal *et al.* (2015)	RCT	Nutritional			
Formanoy *et al.* (2016)	RCT	Physical activity			
Michishita *et al.* (2017)	RCT	Physical activity			
Saavedra *et al.* (2021)	Quasi exp	Physical activity			
Dollard *et al.* (2014)	Quasi exp	Stress management			
Hasson *et al.* (2010)	RCT	Stress management			
Lloyd *et al.* (2017)	RCT	Stress management			
Munz *et al.* (2001)	Pre/Post	Stress management			
Formanoy *et al.* (2016)	RCT	Social environmental			
Evidence synthesis (overall score)^*^			Sufficient evidence (9/13; 69%)	Insufficient evidence (2/11; 18%)	Moderate evidence (3/7; 43%)
	Significant improvement		Non-significant change or inconsistent results		Significant negative effect

^*^Evidence synthesis score: adapted from Hoogendoorn *et al.* (2000). [Sufficient evidence: score >50%; moderate evidence: score of >25 and <50%; insufficient evidence: score <25%].

^a^Depression, anxiety, stress, burnout, mood, fatigue, emotional exhaustion, psychological strain, depersonalization, and psychological distress.

^b^Productivity, morale, sickness absence, need-for-recovery (NfR), work ability, job independence, job performance, job strain, and job attitudes.

^c^Mindfulness, wellbeing/physical health, self-compassion, energy levels, sleep, cognitive strain, self-esteem, personal relationships, and social support.

All 3 studies employing behavioural interventions reported significant improvements in mental health outcomes of depression and the mental health domains of the SF-36 (Kojima *et al.*, 2010; Das *et al.*, 2019, 2020). The rest of the interventions that were effective in improving mental health varied from educational interventions on coping with strain (Saelid *et al.*, 2016), mindfulness (Aikens *et al.*, 2014), nutrition (Agarwal *et al.*, 2015), and to stress management (Munz *et al.*, 2001; Hasson *et al.*, 2010). For the other outcomes of interest and types of intervention, no clear pattern of effectiveness emerged (Table 3). Heterogeneity meant that it was not possible to assess the strength of the effect, conduct a meta-analysis, or assess the effectiveness of specific intervention components on our target behaviours.

## Discussion

Our review identified an array of workplace group-level interventions to improve the mental health of office-based workers in high-stress and low-autonomy jobs in high-income countries. Almost all interventions included a training session or workshop for intervention delivery, several had delivery manuals, but theories of change were rare. The types of interventions found vary, from traditional stress management aiming to change individual behaviour to interventions applied on an organizational level to change the social environment or job design. Most workplace interventions did not use participatory approaches to involve employees in intervention development, implementation and evaluation, and challenges and facilitators were not commonly reported. The findings show evidence of the benefits of group-level workplace interventions for the mental health and well-being of workers in office-based jobs with these characteristics. Overall, there is sufficient evidence for improvements in mental health outcomes (e.g. depression, anxiety, fatigue), and less so for improvements in work-related (e.g. productivity, sickness absence) and other outcomes (e.g. mindfulness, social support). All studies employing behavioural interventions reported significant improvements in mental health outcomes, while no clear pattern of effectiveness was observed for the work or other outcomes of interest and types of interventions employed. Challenges and barriers to successful implementation, engagement, and delivery of these interventions, included changing conditions within workplaces, long/longer durations of interventions, and the flexibility to enable participation.

Our findings of sufficient evidence of the benefits of workplace group-level interventions on the mental health of workplace interventions are in line with previous reviews (Kaspin *et al.*, 2013; Joyce *et al.*, 2016; Hesketh *et al.*, 2020). Recent systematic reviews examining the effectiveness of workplace mental health interventions and organizational and group-level interventions across occupations showed that they can improve several mental health and wellbeing outcomes (Hesketh *et al.*, 2020; Fox *et al.*, 2022). Specifically, workplace group-level interventions can be used effectively to achieve positive change in employee wellbeing (Demou *et al.*, 2018; Fox *et al.*, 2022). Similarly, our review which is focused on a specific worker group, found that the interventions improved several mental health outcomes, including depression, anxiety, stress, and emotional exhaustion. Like previous reviews (Joyce *et al.*, 2016; Demou *et al.*, 2018; Hesketh *et al.*, 2020; Fox *et al.*, 2022), we also found a large variation of interventions, intervention components, and how mental health and well-being were conceptualized, and measured. This created a challenge in being able to reach conclusive results on whether certain types of interventions were effective and for which outcomes in this workforce. In our review, however, all studies employing behavioural interventions reported significant improvements in mental health outcomes. Joyce *et al.* (2016) similarly reported that stronger evidence was provided for CBT-based stress management than for other prevention interventions, although the only outcomes of interest in their review were depression and anxiety (Joyce *et al.*, 2016).

Our scoping review has several strengths. First, it addresses the knowledge gap in contributing to the evidence base workplace interventions for a specific group of workers with significant psychosocial risk factors by unpacking group-level intervention evidence for jobs with similar characteristics more thoroughly. Furthermore, we examined stakeholder involvement and level of participation in intervention design, implementation and evaluation (Skivington *et al.*, 2021). We have consulted a wide range of databases across the medical, public health, and social science disciplines and have identified studies over a long period. Additionally, the findings analyse a relatively large number of employees, with studies from across four continents, which can help identify differences in working practices or challenges and opportunities that may differ across countries. Our review covers many workplaces where people work in high demand, office-based jobs with low autonomy, including administrative jobs across many corporate and public sectors, IT, and call centres. Furthermore, not limiting our intervention design solely on mental health interventions allowed us to identify other pathways/mechanisms of change that have been used in this population that can target mental health either as a primary or secondary intervention outcome. As our review was open to any type of intervention that could target mental health, we identified several different types of interventions. We examined various interventions that focus on factors that impact individual and workplace outcomes.

While this approach allowed us to explore how health issues are addressed by organizations and was a study strength, it also creates some challenges. For instance, we were unable to tease out relative contributions of group-level interventions in multi-component interventions which also included individually targeted activities. Another limitation is the variability, and reliability of the reporting on the implementation process and duration of each intervention. Specific details on stakeholder engagement and participatory approaches were limited. Understanding the implementation process and the time commitment both from an organizations and participants input is necessary to assess feasibility and transferability of interventions or intervention design to other sectors and workplaces. Eligible studies were from high and medium income countries. Low income countries were excluded to limit variability of contextual factors. In the end all our studies were from-high income countries, and this may limit its transferability to workplaces in middle and low income countries, where health and safety regulations, resources, and workplace organization and culture may be very different. Few studies conducted process evaluations, e.g. fidelity, acceptability, and discussions of facilitators and challenges were therefore limited. In this review we only included workplaces with employees working in high strain, office-based jobs with low autonomy. However, in some cases this was not clear if all employees in the study worked in these jobs, or it was not straightforward to assess. In these cases, the reviewers discussed each paper and reached consensus as to whether the study would be eligible or not. Therefore, it is possible that some participants may not strictly adhere to our job criteria. Most interventions were delivered in person with some complimentary online components. This may limit our insights relating to digital/remote delivery of such interventions, which may be more in the current post-pandemic working context where hybrid working is becoming a new norm. Our evidence synthesis was based on a wide range of measures for each outcome. While this is informative, further systematic reviews and meta-analysis are needed to evaluate each outcome measure.

The novelty of our scoping review is that it provides a more nuanced understanding of group-based approaches used in the context of high strain and low autonomy jobs, the challenges and opportunities that exist and the degree of stakeholder involvement in intervention design, implementation and evaluation. We have identified a “menu” of candidate group-level workplace interventions and intervention components (organizational, relational and individual components) that can be used to improve the mental health and well-being of office-based employees in jobs with high strain and low autonomy. These findings can be used across workplaces and countries to assess applicability whether a workplace wishes to be proactive in implementing an intervention to promote wellbeing or reactive to occupational health and safety regulations. Specifically, the findings showed that all interventions included multiple components, almost all included training sessions or workshops, multiple and complimentary delivery modes were used (e.g. online and in-person), and intervention manuals, reminder systems and feedback processes were also routinely used. We found strongest evidence for behavioural interventions targeting mental health outcomes. Understanding the types of interventions, how they are implemented, employee engagement and impact on the desired outcomes can help inform the development of a theory of change for the development of other mental health intervention(s) for similar workplaces and employees. However, few intervention studies include detail on these and this is holding back knowledge and progress on best practice in the delivery and implementation of successful workplace interventions.

## Supplementary Material

wxae012_suppl_Supplementary_Tables

## Data Availability

Data extracted from the original papers is available in the Supplementary Material
