# Supplementary material for: Group-level workplace interventions to improve mental health in low control, high-demand office-based jobs. A scoping review
Source: Ann Work Expo Health. 2024 Mar 15;68(4):335–50. doi: 10.1093/annweh/wxae012 (PMC11033569; doi:10.1093/annweh/wxae012)
Supplement: wxae012_suppl_Supplementary_Tables [file wxae012_suppl_supplementary_tables.pdf]

Group-level workplace interventions to improve mental health in low control, high demand office-based jobs. A scoping review

Supplementary Information

Evangelia Demou[1]*, Carolyn Blake[1], Charisse Tan Llorin[2], Maria Guadalupe Salanga[2], Nino Jose Mateo[3], Ruth Lewis[1], Kirstin R Mitchell[1]

1. MRC/CSO Social and Public Health Sciences Unit, Institute of Health and Wellbeing, University of Glasgow, Glasgow, UK
2. Department of Psychology, De La Salle University, Manila, Philippines
3. Department of Counselling and Educational Psychology, De La Salle University, Manila, Philippines

*Corresponding author:

Dr Evangelia Demou

MRC/CSO Social and Public Health Sciences Unit

University of Glasgow

Berkeley Square, 99 Berkeley Street

Glasgow, G3 7HR

Email: evangelia.demou@glasgow.ac.uk

Group-level workplace mental health interventions

Search Strategies

Database: **Ovid MEDLINE(R)** 1946 to March Week 3 2021
Search Strategy: 14th April 2021

1	white collar.ab,ti.	1721
2	Workplace/	24261
3	((employee* or worker*) adj2 workplace).ab,ti.	379
4	Call Centers/	88
5	(Contact centres or contact centers).ab,ti.	35
6	(low autonomy adj1 work).ab,ti.	2
7	(change or modification or promotion or intervention* or effect* or switch or altering or evaluation or prevention or program?e).ab,ti.	8330511
8	(mental health adj2 (white collar or business or office or workplace or worker or employment or occupation*)).ab,ti.	756
9	(mental illness adj2 (white collar or business or office or workplace or worker or employment or occupation*)).ab,ti.	57
10	(mental disorder adj2 (white collar or business or office or workplace or worker or employment or occupation*)).ab,ti.	5
11	mental health/	42423
12	depression/	125662
13	(wellbeing or well-being).ab,ti.	78661
14	Anxiety/	85874
15	Stress, Psychological/	124369
16	Burnout, Psychological/	668
17	(employee adj2 (stress or mental health or wellbeing or wellness or burnout or anxiety)).ab,ti.	385
18	(job adj2 (anxiety or control or strain* or stress or demand*)).ab,ti.	5635
19	2 or 3 or 4 or 5 or 6	24567
20	8 or 9 or 10 or 11 or 12 or 13 or 14 or 15 or 16 or 17 or 18	385573
21	7 and 19 and 20	2658
22	(group adj2 (process or method* or based or approach* or intervention*)).ab,kf,ti.	59766
23	(participatory adj2 approach*).ab,kf,ti.	1863
24	((participatory or group*) adj2 approach*).ab,kf,ti.	5749
25	(Patient and Public Involvement).ab,kf,ti.	548
26	(patient adj1 public involvement).ab,kf,ti.	19

27	mapping.ab,kf,ti.	151400
28	22 or 23 or 24 or 25 or 26 or 27	214031
29	limit 21 to yr="2000 -Current"	2568
30	21 and 28	115
31	limit 30 to yr="2000 -Current"	114

PsycINFO (EBSCO)

#	Query
S21	S15 AND S19 Limiters - Published Date: 2000 - current
S20	S15 AND S19
S19	S16 AND S17 AND S18
S18	S9 OR S10 OR S11 OR S12 OR S13 OR S14
S17	S7 OR S8
S16	S1 OR S2 OR S3 OR S4 OR S5 OR S6
S15	TI ((group N2 (process or method* or based or approach or intervention*))) OR AB ((group N2 (process or method* or based or approach or intervention*)))
S14	TI ("call centre" or "call center") OR AB ("call centre" or "call center") OR TI ("contact center" or "contact centre") OR AB ("contact center" or "contact centre")
S13	DE "Job Characteristics"
S12	TI (worker* or employee*) N2 workplace OR AB (worker* or employee*) N2 workplace
S11	TI "low autonomy work*" OR AB "low autonomy work*"
S10	TI ("office workers" or "office work" or "office settings" or "desk work*") OR AB ("office workers" or "office work" or "office settings" or "desk work*" or "office employees")
S9	TI ("white collar jobs" or "white collar worker*") OR AB ("white collar jobs" or "white collar worker*")
S8	DE "Workplace Intervention"
S7	TI ((change or modification or promotion or intervention* or effect* or switch or altering or evaluation or prevention or program?e).) OR AB ((change or modification or promotion or intervention* or effect* or switch or altering or evaluation or prevention or program?e).)
S6	TI (job N1 (strain or control or demand)) OR AB (job N1 (strain or control or demand))
S5	TI ((employee N2 (burn out or burnout or burn-out)) OR AB ((employee N2 (burn out or burnout or burn-out))
S4	TI "employee mental health" OR AB "employee mental health"
S3	TI (mental illness or mental health or mental disorder) OR AB (mental illness or mental health or mental disorder)

S2	AB (stress or wellness or wellbeing or well-being or well being) N1 (employee) OR TI (stress or wellness or wellbeing or well-being or well being) N1 (employee)
S1	(((DE "Occupational Stress") OR (DE "Depression (Emotion)")) OR (DE "Mental Health")) OR (DE "Anxiety")

CINAHL (EBSCO)

#	Query
S25	S22 AND S23 Published Date: 2000 - current
S24	S22 AND S23
S23	S7 AND S8 AND S21
S22	S15 OR S16 OR S17 OR S18 OR S19 OR S20
S21	S9 OR S10 OR S11 OR S12 OR S13 OR S14
S20	TI mapping OR AB mapping
S19	TI patient N1 "public involvement" OR AB patient N1 "public involvement"
S18	TI ("Patient and Public Involvement") OR AB ("Patient and Public Involvement")
S17	TI ((participatory or group*) N2 approach*) OR AB ((participatory or group*) N2 approach*)
S16	TI participatory N2 approach* OR AB participatory N2 approach*
S15	TI ((group N2 (process or method* or based or approach or intervention*))) OR AB ((group N2 (process or method* or based or approach or intervention*)))
S14	TI ("call centre" or "call center") OR AB ("call centre" or "call center") OR TI ("contact center" or "contact centre") OR AB ("contact center" or "contact centre")
S13	(MH "Job Characteristics") or (MH "Work Environment")
S12	TI (worker* or employee*) N2 workplace OR AB (worker* or employee*) N2 workplace
S11	TI "low autonomy work*" OR AB "low autonomy work*"
S10	TI ("office workers" or "office work" or "office settings" or "desk work*") OR AB ("office workers" or "office work" or "office settings" or "desk work*" or "office employees")
S9	TI ("white collar jobs" or "white collar worker*") OR AB ("white collar jobs" or "white collar worker*")
S8	TI ((change or modification or promotion or intervention* or effect* or switch or altering or evaluation or prevention or program?e).) OR AB ((change or modification or promotion or intervention* or effect* or switch or altering or evaluation or prevention or program?e).)
S7	S1 OR S2 OR S3 OR S4 OR S5 OR S6
S6	TI (job N1 (strain or control or demand)) OR AB (job N1 (strain or control or demand))
S5	TI ((employee N2 (burn out or burnout or burn-out)) OR AB ((employee N2 (burn out or burnout or burn-out))

S4	TI "employee mental health" OR AB "employee mental health"
S3	TI (mental illness or mental health or mental disorder) OR AB (mental illness or mental health or mental disorder)
S2	AB (stress or wellness or wellbeing or well-being or well being) N1 (employee) OR TI (stress or wellness or wellbeing or well-being or well being) N1 (employee)
S1	(MH "Mental Health") or (MH "Depression") or (MH "Anxiety")

ASSIA (Proquest)

(noft(mental illness NEAR/2 (worker OR employment OR occupation)) OR noft(mental health NEAR/2 (worker OR employment OR occupation)) OR noft(mental disorder NEAR/2 (worker OR employment OR occupation)) OR (MAINSUBJECT.EXACT("Depression") OR MAINSUBJECT.EXACT("Anxiety-Depression")) OR MAINSUBJECT.EXACT("Mental health") OR (MAINSUBJECT.EXACT("Emotional wellbeing") OR MAINSUBJECT.EXACT("Psychological wellbeing") OR MAINSUBJECT.EXACT("Wellbeing")))

AND (noft((employee* OR worker*) NEAR/2 workplace) OR MAINSUBJECT.EXACT("Workplaces") OR noft((call center* OR call centre*)) OR noft((contact centre* OR contact center*)) OR noft(white collar) OR noft(office based))

Table S1. Full descriptive table of included studies

Study ID	Country	Design	Setting<br>Type of intervention<br>Participatory Approach in intervention development (design), implementation and/or evaluation	Participants/ Sample size	Target outcomes (measurement tool)	Intervention description
Agarwal et al. 2015[1]	USA	RCT (Quasi-experimental study)–	10 corporate sites of an insurance company<br>Nutritional Intervention	Employees: Total n =292<br>Intervention: n = 142<br>Control: n =150<br>Gender:	<u>Primary</u><br>Depression (SF-36)<br>Anxiety (SF-36)<br><u>Secondary</u><br>Work productivity (WPAI)	*<u>Intervention delivery</u>*<br>• Online & in-person; Office based delivery<br>• 18 weeks<br>• Mixed group-level & individual components<br>*<u>Intervention components</u>*

			Participatory approach:<br>Not reported	Male : 20.2%<br>Female: 79.8%<br>Intervention group: 31<br>Control group: 18<br><br>Age:<br>43.84 ±10.61 (Int)<br>45.38 ± 11.32 (cont)		• Group instruction & support during weekly lunch hour classes at the worksite<br>• Classes led by registered dietician, physician,<br>• and/or a cooking instructor<br>• Nutrition education lectures<br>• Cooking demonstrations<br>• Interactive online message board<br>• Low-fat vegan menu options at workplace cafeteria (where available)<br>• Gift voucher (for control group ONLY)
Ahola et al. 2012[2]	Finland	Randomised Controlled study	17 organisations<br>-9 city administration<br>-5 government organisations (2 city department; research institute; employment & insurance office)<br>-3 private companies	Employees: Total n = 566<br><br>Intervention: n = 296<br>Control: n = 270<br><br>Gender:<br>Male 11%	<u>Primary</u><br>Depression<br><br>Secondary<br>Job Strain<br>Depressive symptoms	*<u>Intervention delivery</u>*<br>• In-person; Workplace & non-workplace delivery<br>• Training sessions delivered by two trainers who were also employees (one from occupational health services and one form human resources). The two employees from each organization were trained at the Finnish Institute of Occupational Health (non-workplace)

			(banking, occupational health service, multiservice company)<br><br>Skills training intervention (to enhance participants' career management preparedness by strengthening self-efficacy and inoculation against setbacks) (education – group training)<br>Skills training used active learning methods.<br><br>Participatory approach:	Female: 89%<br><br>Age: 50 ± 6.5		• Main intervention: 4 half-day session, delivered over 1 or 2 weeks (workplace, delivered by the two trainers)<br>• Group-level<br><br>*Intervention components*<br>• Intervention manual<br>• Skills training workshops<br>• Trainers (occupational health & human resources)<br>• Literature package on career management and health related information (Control group only)

			-implementation<br>(trainers)			
Aikens et al. 2014[3]	USA	Randomized Controlled Study design	Chemical industry<br>Mindfulness Intervention<br>Participatory approach:<br>-implementation	Employees: Total n = 90<br>Intervention: n = 44<br>Control: n = 45<br>Gender:<br>Not reported<br>Age: not given	<u>Primary</u><br>Stress<br>Mindfulness<br>Wellbeing	*<u>Intervention delivery</u>*<br>• Online & in-person; Workplace & non-workplace delivery<br>• 7 weeks (follow-up @ 6 months for intervention group)<br>• Mixed Both Group-level & individual components<br>*<u>Intervention components</u>*<br>• Unique training online dashboard<br>• Virtual classes & accompanied online applied training<br>• Online weekly progress tracking survey<br>• Tailored email coaching and feedback<br>• Customised text messaging system (daily reminder, progress update & encouragement)
Arrendondo et al. 2017[4]	Spain	RCT	Private international clinical research organization company	Employees: Total n = 40<br>Intervention: n = 21	<u>Primary</u><br>Stress	*<u>Intervention delivery</u>*<br>• In person; Workplace delivery

			Mindfulness<br>Intervention<br><br>Participatory approach:<br>Not reported	Control: n = 19<br><br>Gender:<br>Male 22.5%<br>Female: 77.5%<br><br>Age: 36.6 ± 5.6	<u>Secondary</u><br>Mindfulness<br>Heart Rate<br>Variability<br>Self-compassion<br>Decentering<br><br>Burnout	• 8 weeks (follow-up @ 20 weeks for intervention group)<br>• Mixed: Group-level with individual components (formal daily practice)<br><br>*<u>Intervention components</u>*<br>• Brief integrated practices<br>• 8 x 1.5 hrs training sessions<br>• Mindfulness retreat (3 hrs)<br>• Formal daily practice sessions (12-16 mins)<br>• Daily record dairy
Das et al. 2019[5]	USA	RCT	12 Broad range of worksites (Intervention: 8 worksites - 4 universities, 3 for profit companies, 1 non-profit organization; Control: 4 worksites- 1 university, 2 for-profit companies,	Employees: Total n = 240<br><br>Intervention: n = 163<br>Control: n = 77<br><br>Gender:<br>Male 41.7%	<u>Primary</u><br>Employee vitality (Energy)<br><br><u>Secondary</u><br>Quality of Life<br>Purpose in Life<br>Sleep	*<u>Intervention delivery</u>*<br>• Online & in-person; Workplace & non-workplace delivery<br>• 2.5 days (follow-up @ 6, 12 & 18 months)<br>• Group-level<br><br>*<u>Intervention components</u>*<br>• 3 x Trained coaches

			and 1 nonprofit organization.<br>Behavioural intervention<br>Participatory approach: -implementation	Female: 58.3%<br><br>Age: 46	Mood<br>Depression<br>Body Mass Index	• Supplemental educational materials (e.g. workshop manual, portable exercise booklet with quick, energizing workouts)<br>• Comprehensive online support (e-course) available for the entire follow-up period
Das et al. 2020[6]	USA	Pre-post intervention analysis	Broad range of worksites (e.g. 5 universities, 5 for-profit companies, and 2 non-profit organizations)<br>Behavioural intervention<br>Participatory approach: Not reported	Employees: Total n = 240<br><br>Intervention: n = 163<br>Control: n = 77<br><br>Gender:<br>Male 41.7%<br>Female: 58.3%<br><br>Age: 46.5±10.8	<u>Primary</u><br>Vitality<br>PiL<br><br><u>Secondary</u><br>Sleep<br>Mood<br>Depression<br>BMI	*<u>Intervention delivery</u>*<br>• Online & in-person; Non-workplace delivery<br>• 2.5 days (follow-up @ 12 & 18 months)<br>• Group-level<br><br>*<u>Intervention components</u>*<br>• Commercially available intervention<br>• Trained coaches<br>• In-person Interactive sessions<br>• Comprehensive online support (e-course)<br>• Participants design their own 'action plan' to sustain change post-intervention.

Dollard et al. 2014[7]	Australia	Quasi-experimental cohort study	Public sector organisation<br><br>Stress Management intervention<br><br>Participatory approach: -design	Employees: Total<br>n (T1) = 605<br>n (T2) = 679<br><br>Intervention:<br>n (T1) = 94<br>n (T2) = 123<br><br>Control:<br>n (T1) = 511<br>n (T2) = 556<br><br>Gender at T1:<br>Male: 54%<br>Female: 46%<br><br>Age: not given	Primary<br>Organisational & job design factors<br><br>Secondary<br>Stress<br>Morale<br>Sickness Absence<br>Duration	*Intervention delivery*<br>• In person; Workplace delivery<br>• 4 x 4 hrs/week<br>• Group-level<br><br>*Intervention components*<br>• Educational workshops<br>• External expert in organisational psychology<br>• Facilitator guidance material for standardised approach<br>• Risk assessment & routine data were used for intervention development

Formanoy et al. 2016[8]	The Netherlands	Cluster RCT	Financial service provider<br><br>Social environmental & physical activity intervention<br><br>Participatory approach: Not reported	Employees: Total n = 312<br><br>Intervention:<br>n = 149 & 132<br>Control:<br>n = 163 & 180<br><br>Gender:<br>Male: 60%<br>Female: 40%<br><br>Age: range 19 -63yrs	<u>Primary</u><br>Need for Recovery (NFR after work scale)	<u>*Intervention delivery*</u><br>• Online & in-person; workplace delivery<br>• Duration: not clear (follow-up @ 12 months)<br>• Mixed group-level & individual components<br><br><u>*Intervention components*</u><br>• Social environmental component<br>o Motivational interviewing sessions<br>o Trained team leaders<br>o Wed-based social media platform<br>• Physical environmental component<br>o Changing (social) 'coffee corners'<br>o Environmental changes to open office environment, meeting rooms, entrance (e.g. exercise balls, standing tables, table tennis, footsteps to promote stair use)
Grégoire, and	Canada	Pretest-post-test	Call centre in a financial service provider	Employees: Total n = 49	<u>Primary</u><br>Mindfulness (MAAS)	<u>*Intervention delivery*</u><br>• In-person; workplace delivery

Lachance. 2015[9]		control group switching-replication design	Mindfulness intervention<br><br>Participatory approach: Not reported	Intervention: n = 24 (25 @ T2)<br>Control: n = 25 (24 @ T2))<br><br>Gender:<br>Male 8%<br>Female: 92%<br><br>Age: 35.8 (range: 26-57 yrs)	Stress (PSM-9)<br>Anxiety (PDMS)<br>Depression (PDMS)<br>Fatigue (FSS)<br>Negative affects<br><br>Secondary	• 15 mins/day over 5 weeks<br>• Mixed group-level & individual components<br><br>*Intervention components*<br>• Group information meetings @ intervention start and mid-intervention<br>• Daily guided brief body scans & sitting meditation sessions<br>• Audio sessions recorded by experienced mindfulness coach with language tailored to corporate world<br>• Audio sessions available on internal server<br>• Protected time and space during work hours to listen to audio sessions<br>•
Hasson et al. 2010[10]	Sweden	RCT	Information technology<br><br>Stress management intervention	Employees: Total n = 303<br><br>Intervention: n = 129<br>Control: n =174	Primary<br>Stress<br><br>Secondary	*Intervention delivery*<br>• Online; workplace delivery; non-workplace delivery: unclear<br>• 12 months<br>• Mixed group-level & individual components

			Participatory approach:<br>N/A<br>Cognitive ergonomics intervention<br>Participatory approach:<br>-design<br>-implementation<br>-evaluation<br>-monitoring	Gender:<br>Male 62%<br>Female: 38%<br><br>Age:<br>≤ 30: 26%<br>31–45: 38%<br>≥ 46: 36%		*Intervention components*<br>• Static (control & intervention) & Interactive (intervention) web-based programme<br>• Online diary & feedback<br>• Automatic email reminder option<br>• Web-based self-help exercises, viewed in html, downloaded as a PDF or as a Flash animation with picture and sound<br>• Chat room for intervention participants<br>• Feedback on blood samples (linked to incentive for participation)
Kojima et al. 2010[11]	Japan	Controlled trial	Office workers of a metal manufacturing industry	Employees: Total n = 261	Primary<br>Depression (CES-D)<br>Self-esteem (RSES)	*Intervention delivery*<br>• Online & in-person; workplace & non-workplace delivery

			Cognitive Behavioural Therapy Training intervention<br><br>Participatory approach: Not reported	Intervention: n = 137<br>Control: n = 124<br><br>Gender:<br>Male: 70% (Int)<br>Female: 30% (int)<br>Male: 66% (Cont)<br>Female: 34% (Cont)<br><br>Age:<br>33.2 ± 1.75 yrs (Int)<br>33.1 ± 1.71 yrs (Cont)	<u>Secondary</u><br>- an understanding of stress control skills<br>- will to apply these stress control skills (questions drafted for this study).	• 1x 3hrs group session & 3 email sessions (Intervention ran from July to December 2007)<br>• Follow up: 1w and 3m after end of training<br>• Mixed group-level & individual components<br><br>*<u>Intervention components</u>*<br>• Group training delivered by CBT specialist trainers (psychiatrist & psychotherapist)<br>• Personal email sessions ('feedback, advice, directing to further resources) delivered by occupational health staff, who have received training from CBT trainers<br>• Homework assignments<br>• Checklist to aid group session
Lloyd et al. 2017[12]	UK	RCT	Government departments (employees with customer facing roles)	Employees: Total n = 153<br><br>Intervention: n = 68<br>Control: n = 85	<u>Primary</u><br>Psychological strain<br>Emotional exhaustion<br>Depersonalization	*<u>Intervention delivery</u>*<br>• In-person; workplace & non-workplace delivery<br>• 2 x 3 hr training session over two weeks & a 3 hr training session 2 months after

			CBT-focused Stress management intervention<br><br>Participatory approach: Not reported	Gender:<br>Male: 21%<br>Female: 79%<br><br>Age: 46.2yrs<br>(range: 19–63yrs)		• Follow up: 6m after final training (outcome measures also measured 2m after training)<br>• Mixed group-level & individual components<br>*Intervention components*<br>• Protected time for intervention participation (during work hours)<br>• Onsite (conference rooms)<br>• Support materials included homework assignments, handouts, training session summary sheets and CD's
Michishita et al. 2017[13]	Japan	Randomised controlled study	White-collar<br><br>'Active rest' intervention (physical activity)<br><br>Participatory approach: Not reported	Employees: Total n = 59<br><br>Intervention: n = 29<br>Control: n =30<br><br>Gender:<br>Male 66% (Int)<br>Female: 34% (Int)<br>Male 70% (Cont)	Primary<br>Personal relationships,<br>Profile of Mood States<br>Physical activity<br>Physical health<br>Work ability	*Intervention delivery*<br>• In-person; workplace delivery<br>• 10 weeks (10 mins per day, 3 x week)<br>• Group-level active rest program<br>*Intervention components*<br>• Commercially available fitness programme<br>• Fitness instructor<br>• Onsite (conference room, could not be observed by others)

				Female: 30% (Cont)<br><br>Age:<br>40.8 ± 9.8 yrs (Int)<br>41.1 ± 8.6 yrs (Cont)	<u>Secondary</u>	• 10 min sessions include warm-up (stretching), cognitive functional training, aerobic exercise, body weight resistance training and cool-down<br>• Anthropometry and blood pressure measurements<br>• Physical activity measurement (accelerometer)
Mills et al. 2007[14]	UK	Quasi-experimental pre-post intervention-control study	Office based employees of a multinational manufacturer of food, home care, and personal care products<br><br>Health promotion intervention<br><br>Participatory approach: Not reported	Employees: Total n =<br><br>Intervention: n = 519 (@ baseline); n=266 (@ follow-up)<br>Control: n = 1679 (n=1242 at follow up)<br><br>Note: control group described as "recruited from the community" (i.e. unclear if they were	<u>Primary</u><br>Count of health risk factors<br>Sickness absence<br>Work performance<br><br>Secondary<br><br>Return on investment due to health risk change	*<u>Intervention delivery</u>*<br>• Online & in-person; workplace delivery<br>• 12 months follow up<br>• Mixed group-level & individual components<br><br>*<u>Intervention components</u>*<br>• Intervention program via e-mail communication<br>• Personalized health and well-being report<br>• Tailored advice on improvement of personal health<br>• Unlimited web portal access (articles, assessments, interactive online behaviour-change programs)<br>• Tailored fortnightly emails

				employees of the organisation)<br><br>Gender:<br>Male 46% (Int)<br>Female 54% (Int)<br>Male 46% (Int)<br>Female 54% (Cont),<br>unweighted; 54% weighted<br><br>Age:<br>35.2 yrs (Int)<br>41.9 yrs (Cont)	Intervention impact on individual health risk factors	• Paper-based information packs (pg. newsletter and health promotion literature)<br>• On-site wellness seminars
Munz et al. 2001[15]	US (?)	Pre-post, treatment-control	Telecommunications company (customer service/sales representatives) in 4 different cities	Employees: Total n = 79<br><br>Intervention: n = 55<br>Control: n = 24	<u>Primary</u><br>Individual level:<br>- Emotional wellbeing (perceived stress,	<u>*Intervention delivery*</u><br>• In-person; workplace delivery<br>• 4 x 3 hr modules<br>• Mixed group-level & individual components

			Worksite stress management program<br><br>Participatory approach: Not reported	Gender:<br>Male (-)<br><br>Age: (-)	positive and negative affect, depression)<br><br>Organisational level:<br>Work group productivity<br>Sickness absence<br><br><u>Secondary</u><br>Job independence	*<u>Intervention components</u>*<br>• Individual stress management components<br>o Seminars, self-assessments, skills training<br>o Voluntary component<br>o A participant manual<br>• Organisational stressor reduction process<br>o Workshop facilitator (psychologist), development of action plan and evaluation<br>o Feedback and recommendation loops implemented<br>o Routine company data used
Saavedra et al. 2021[16]	Iceland	Quasi-experimental (semi-randomized; pilot study)	Sedentary office work setting<br><br>2 physical activity interventions: circuit	Employees: Total n = 47<br><br>Intervention:<br>n (CT) = 18<br>n (BW) = 18<br>Control:	<u>Primary</u><br>Anthropometry<br>Body composition<br>Cardiorespiratory fitness<br>Lipid profile	*<u>Intervention delivery</u>*<br>• In-person; workplace delivery<br>• 3 x 30 min session per week, over 12 weeks<br>• Group-level<br>*<u>Intervention components</u>*

			training (CT); brisk walk (BW)<br><br>Participatory approach: Not reported	n = 11<br><br>Gender:<br>Male: 27%<br>Female: 73%<br><br>Age: 45 ± 11.95 yrs	Blood pressure<br>Mental health (depression, anxiety, stress)<br><br>Secondary	• Protected worktime to participate in intervention (i.e. extra 15 min added to lunch break)<br>• Facilitator from research team<br>• CT intervention: Practice equipment (resistance bands, free weights, aerobic steppers, medicine balls, bosu balls, and exercise mats)<br>• BW intervention: Walking sessions around workplace
Saelid et al. 2016[17]	Norway	RCT	Public organisations<br><br>Coping with strain intervention (educational?)<br><br>Participatory approach: Not reported	Employees: Total n = 119<br><br>Intervention Grp I: n = 59<br>Intervention Grp II: n = 60<br><br>Gender: Not reported<br><br>Age:	Primary<br>Burnout<br>Depressive symptoms<br>Self-efficacy<br>Quality of life<br>Self-esteem<br>Social support<br>Negative life events<br>Sickness absence<br><br>Secondary	*Intervention delivery*<br>• In-person; workplace & non-workplace (home) delivery<br>• 8 x 2.5 hr/week & 2 booster sessions 1 or 2 months after (follow-up @ 8 months & 3 times over next 3 years)<br>• Group-level<br>*Intervention components*<br>• CWS work manual for home assignments<br>• Standardized manual for course leaders & textbook for participants<br>• Delivered by a trained therapist and assistant

				Intervention Grp I:<br>41-50 32%<br>51-60 34%<br>Intervention Grp II:<br>41-50 34%<br>51-60 33%		
Smith. 2008[18]	Australia	RCT	Call centre – customer facing employees<br>Music relaxation intervention<br>Participatory approach: Not reported	Employees: Total n = 80<br>Intervention: n = 40<br>Control: n = 40<br>Gender:<br>Male 50%<br>Female: 50%<br>Age: 37.5 ± 1.9 yrs	Primary<br>Anxiety (STAI)<br>Secondary	*Intervention delivery*<br>• In-person; workplace delivery<br>• 15 min<br>• Group-level<br>*Intervention components*<br>• Facilitator (research team member) for both intervention and control<br>• Live improvised guitar music<br>• Progressive Muscle Relaxation (PMR) exercises<br>• Control: participant-led discussion on experiences & distressing call during shift

Group-level workplace mental health interventions

Takao et al. 2006[19]	Japan	RCT	Sake brewery<br><br>Job stress education intervention delivered to supervisors<br><br>Participatory approach: Not reported	Employees:<br>Total n =301<br>Supervisors: 46<br>Subordinates: 255<br><br>Intervention: n=24<br>Supervisors: n = 24<br>Subordinates: n =154<br>Control: n = 22<br>Supervisors: n = 22<br>Subordinates: n =101<br><br>Gender of "subordinates"<br>Male: 66.4% (Int)<br>Female: 33.6% (Int)<br>Male: 69.6% (Cont)<br>Female: 30.4% (Cont)	<u>Primary (measures of subordinates, not supervisors)</u><br>Psychological distress<br>Job performance<br><br><u>Secondary</u>	*<u>Intervention delivery</u>*<br>• In-person; workplace delivery<br>• 2 sessions (1st: 60 min & 2nd: 180 min)<br>• Follow up 3m<br>• Group-level<br><br>*<u>Intervention components</u>*<br>• Educational program for supervisors<br>○ Standardized protocol & intervention manuals<br>○ Handouts<br>○ Facilitators: occupational physician & psychologist<br>• Active listening training session for supervisors<br>○ Trainers: clinical psychologists<br>○ Lecture and role-playing exercise

				Age (Int/Cont):<br>≥50 yrs: 31.3/23.9%<br>35–49 yrs: 20.9/14.1%<br>≤34 yrs: 47.8/62.0%		
Workman and Bommer. 2004[20]	USA	Randomly assigned pre-test/ post-test and control group design	Call centre of an international computer company<br><br>Job redesign – comparison of 3 types of intervention<br><br>Participatory approach: Not reported	Employees: n = 149<br><br>Intervention:<br>n=35 (RA)<br>n=43 (HIWP)<br>n=35 (Autonomous)<br>Control: n = 36<br><br>Gender:<br>Male: 83%<br>Female: 17%<br><br>Age: 31.07 ± 7.14 yrs	<u>Primary</u><br>Job strain<br>Job attitudes<br><br><u>Secondary</u>	<u>*Intervention delivery*</u><br>• In-person; workplace delivery<br>• Duration: unclear<br>• Group-level<br><br><u>*Intervention components*</u><br>• Intervention 1: Alignment job design (AJD):<br>○ Examine performance measurements & outcomes; adjust them according to strategic organisational objectives; adapt reward system (merit pay and bonuses) based on meeting aligned goals<br>○ 'Hot seat' roles/responsibilities rotation

						• Intervention 2: High-involvement performance work process (HIWP)<br>○ Job redesign<br>○ Gainsharing reward system added to individual-based merit pay<br>○ Training seminar<br>○ 'Lunch and learn participation workshops'<br>• Intervention 3: Autonomous work teams (AWT)<br>○ Management relinquishes control- Team members developed written work agreements<br>○ Team-based measurement and rewards<br>○ 'Hot seat' roles/responsibilities rotation

Table S2. Detailed barriers, facilitators and recommendations for group-based mental health interventions

Study ID	Challenges	Facilitators	Recommendations
Agarwal et al. 2015[21]	• Self-selection bias and participant representativeness issues<br>• Quasi-randomised study: workplace randomised not employees<br>• Control group did not receive any intervention; however the presence of an intervention could account for the observed effects	• A study design can be easily implemented in other corporate locations	
Ahola et al. 2012[2]	• Representativeness of participating workplaces & employees could not be assessed<br>• Participating organisations: large- & and medium-sized, in urban areas, predominantly public sector, and dominated by female workers (75%)<br>• Stratified analyses may have suffered from diminished statistical power<br>• Could not control for important factors, including psychological contract, psychological capital, and psychological involvement with work among the participants<br>• A detailed cost and benefit analysis could not be performed<br>• Depression assessed based on self-report using a scoring tool.<br>• Participants with prior illness were excluded (representativeness?)	• Theory-based intervention, delivered in a structured way strictly according to a published manual.<br>• Intervention was delivered by the organisation's own employees, with a combination of health-care professionals (occupational healthcare) and lay personnel (human resources)<br>• Results can be carefully generalised to similar settings in which the participation is voluntary	• More studies on different employee groups are warranted<br>• Future studies should focus on (a) the mediating mechanisms of the intervention; (b) group composition; (c) different implementation procedures; and (d) cost-benefit analysis<br>• Other factors which affect the success of the intervention, should be included in future designs, e.g. income level, psychological contract and capital<br>• Providing more information about the intervention would help wider distribution and testing
Aikens et al. 2014[3]	• Study based on relatively small number of participants<br>• Only self-report measures<br>• Follow-up was not completed to avoid overburdening the employees<br>• Could not control for important factors, including increased attention, being part of a credible treatment program, or social and	(-)	• Future larger randomised control trial is needed to assess effectiveness

	group-related factors, that may have partially contributed to study outcomes<br>• Uncertainty about generalisability of findings<br>•		
Arrendondo et al. 2017[4]	(-)	(-)	• Future research with larger samples must examine the impact of such interventions on costs, long-term productivity, health outcomes, and assess the mechanisms of action of these mindfulness interventions
Das et al. 2019[5]	• Only self-report measures<br>• Potential self-selection bias and participant representativeness issues	(-)	• Future studies of varied duration on this and similar interventions are needed to measure intensity, sustainability, and frequency of delivery and touchpoints, to understand how to maximize participation, cost-effectiveness, and program benefits
Das et al. 2020[6]	• Significant missing data; may impact interpretation of the findings<br>• Representativeness of participating employees could not be assessed<br>• Uncertainty regarding generalisability of findings<br>• Likelihood of attrition in control group may increase in the wait-listed control group design applied<br>• Lack of control group beyond 6 months<br>• Lack of cost-effectiveness analysis	• The health and well-being intervention is commercially available for large-scale dissemination	• Future cost-effectiveness studies are warranted<br>• future studies that evaluate the intervention at the population level should offer equal enrolment opportunity for eligible employees
Dollard et al. 2014[22]	• Using anonymous data means cannot control for within group composition changes (e.g. staff turnover/replacement)<br>• As organisation sought to improve high risk groups, cannot assess generalisability of findings or regression to the mean<br>• Interventions tailored as a variety of issues were covered and activities implemented, making point and mechanisms of change hard to establish	(--)	• Future research is needed with a longer implementation & evaluation time<br>• More effort is warranted in understanding the starting conditions<br>• Validated psychometric outcomes measures are needed<br>• Validation of the value-added of specific measures (in ODS) are required

Formanoy et al. 2016[8]	• Participation engagement (not all participants in the fully participated in the social & physical environmental interventions)<br>• Time and other factors (e.g. holidays) were reported as barriers for participation<br>• Low engagement (i.e. sample size) may impact interpretation of the findings<br>• Not a full RCT (social environmental intervention group was randomized, but the physical environmental intervention was not)	• Advanced exploratory analyses methods (e.g. QUINT) are able to indicate for which subgroups of workers a worksite health program is beneficial and for which subgroups it is not	• A “one size fits all” approach is not suitable; the effectiveness of an intervention may differ across individuals. Worksite wellbeing program developers should consider possibilities to tailor the program to specific subgroups<br>• Age and working overtime should be taken into account when developing a health program for office workers, and also when designing health intervention evaluation studies
Grégoire, and Lachance. 2015[9]	• Possible selection bias; therefore potential impact on the validity of findings<br>• Previous participant experience of mindfulness was not captured (validity)<br>• Small sample size<br>• Short time period<br>• Generalisability issues (sample homogeneity was high)<br>• Assessment tool may not be most appropriate and self-reporting nature of measures can introduce biases<br>• The intervention design, means that employees cannot benefit significantly from the group dynamic, take advantage of personal supervision and support or guidance from the trainer	• Intervention is less time-consuming than typical MBSR programs; busy employees with no prior experience with meditation may be more inclined to try this program and follow through to completion & easier for employers to integrate into regular work schedule<br>• Flexible intervention- majority of mindfulness training occurs at workstation; flexibility convenient for organizations with employees in various, locations, working from home, or that cannot afford to gather employees in training rooms for long periods of time (e.g. call centres)<br>•	• Future research needs to examine to impact of the experienced intervention facilitator; would similar results be obtained with an instructor who had less experience with meditation or even a member of staff?<br>• Further research is warranted on examining to what extent instructors need to be present in the training room (and not online) with their participants in order for the intervention to be effective.<br>• Important to understand to what extent can mindfulness meditation be self-taught<br>• Efforts should also be made to assess the consistency of employee participation in interventions
Hasson et al. 2010[10]	• Chat room component not readily utilised; probably due to too few participants visiting at the same time & limited number of participants in intervention group<br>• Biological markers were used in the present study as health variables; cannot rule out that	(-)	• More research is needed regarding participants education levels in relation to their use of different type of web-based health interventions to enable development of web programs that are tailored<br>• Future studies should focus on web-based health programs potential to motivate and

	biomarker levels may differ between individuals for reasons unrelated to intervention<br>• Participants had access to the intervention from any computer; therefore could not assess the possible influence of the place or time of participation		engage less healthy individuals to participate<br>• More sophisticated measures of participation such as number of page views or average viewing time could have highlighted the participation in the program in more detail<br>• Addition of interactive exercises where participants can learn techniques for improving their health and well-being might increase program utilisation
Kojima et al. 2010[11]	• Frequency of face-to-face contact with specialist is impractical in workplaces<br>• High level in attrition in follow-up questionnaire for both participants and control<br>• Not able to assess several possible confounding factors, including daily life stressors and working hours	• CBT training in the present study was characterized by individualized e-mail sessions provided to each participant following a group session; participants may have been encouraged by the staff's timely response<br>• Group session was conducted by CBT specialists; may have contributed to better understanding of CBT<br>• Learning alongside colleagues may have motivated participants to learn CBT<br>• The positive results suggest that training administered by non-specialists can be effective given appropriate support from CBT specialists	• Research with longer follow-up is needed<br>• Further consideration should be given to the 'correct' balance of the number of group and email sessions required<br>• Larger interventional studies involving participants reflecting a range of ages and occupations are needed to clarify effectiveness
Lloyd et al. 2017[12]	• High attrition rate (affected by work scheduling, travel issues, and sick leave.	(-)	• Future work needs to examine whether certain groups of employees may need additional and/or other forms of development activity may be beneficial (e.g. may be beneficial to attempt to enhance motivation prior to the delivery of SMT or to channel these employees into more individualised forms of development such as coaching or counselling, rather than including them in group training interventions).

			• Organisations may consider attempting to enhance intrinsic motivation more broadly; setting up a work environment that is inherently motivating may be a more proactive way to manage occupational strain (e.g. via effective job design)
Michishita et al. 2017[13]	(-)	(-)	• Further investigation in a larger population, including population of workers in other occupations or with health complications, will be necessary to more precisely clarify the mechanisms underlying this association and the implications for occupational health
Mills et al. 2007[14]	• Outcomes based entirely on self- reports	(-)	• Future research should examine the possibility of including an intervention arm that provides only self-directed tools (e.g. books, videos) to evaluate the relative importance of these materials compared with the group process and tailored elements in the full intervention
Munz et al. 2001[23]	(-)	(-)	(-)
Saavedra et al. 2021[16]	• Due to outdoor activity, the weather caused walking session was cancelled once; however this was compensated for by adding an extra walking session on another day in the same week.	• Programs that utilise employee's lunchtime could be implemented in other companies with this type of a structural timetable	• Studies with larger samples are necessary to assess whether the conclusions can be generalized
Saelid et al. 2016[17]	• Lack of control group; cannot rule out the possibility that other factors may have contributed to the observed long-term reduction in symptoms of depression	(-)	(-)
Smith. 2008[24]	• The researcher was previously employed at this call centre, and knew all the participants involved.	• Knowing the participants created a sense of comfort in the group	
Takao et al. 2006[19]	• Cultural context around age may have influenced intervention; due to traditional	n/a	

	customs in Japanese society, young supervisors might have considered it difficult to advise their older subordinate son work-related matters, and this may have in turn weakened the effect of the supervisor education program on the subordinates.		
Workman and Bommer. 2004[25]	(-)	(-)	Future research needs the assess generalizability of findings and consider if: (i) the results can be replicated in other computer technology-based call centres?; and (ii) can these results be replicated in call centres generally?

Table S3. Effectiveness of interventions

Study ID	Post-intervention
Agarwal et al. 2015[21]	**Mental Health outcomes**<br>*Depression*<br>Intention to treat analysis: Between-Group Difference, mean, adjusted= 3.72 (0.49 to 6.94); p=0.02<br>Completers analysis: Between-Group Difference, mean, adjusted = 6.38 (2.06 to 10.70); p=0.004<br>*Anxiety*<br>• Intention to treat analysis: Between-Group Difference, mean, adjusted = 3.59 (0.25 to 6.92); p=0.04<br>• Completers analysis: Between-Group Difference, mean, adjusted = 5.82 (1.31 to 10.32); p=0.01<br><br>**Work Outcomes**<br>*Overall work impairment because of health*<br>• Intention to treat analysis: Between-Group Difference, mean, adjusted = -0.06 (-0.11 to -0.01); p=0.02<br>• Completers analysis: Between-Group Difference, mean, adjusted = -0.11 (-0.18 to -0.03); p=0.005<br><br>**Other outcomes**<br>*Fatigue*<br>• Intention to treat analysis: Between-Group Difference, mean, adjusted = 7.67 (4.04 to 11.29); p<0.001<br>• Completers analysis: Between-Group Difference, mean, adjusted = 13.50 (8.80 to 18.19); p<0.001<br>*Emotional well-being*<br>• Intention to treat analysis: Between-Group Difference, mean, adjusted = 3.82 (0.89 to 6.75); p=0.01<br>• Completers analysis: Between-Group Difference, mean, adjusted = 6.26 (2.29 to 10.24); p=0.002
Ahola et al. 2012[2]	*Depression*<br>• Seven months after the intervention, the prevalence of potential depression was 8% (n=23) in the intervention group and 12% (n=33) in the comparison group.<br>• The intervention had a statistically significant effect (OR=0.40, 95% CI 0.19 to 0.82, p=0.01) on depression at T2 after depressive symptoms at baseline were adjusted for (model 1);<br>• After additional adjustment for socio-demographic factors and job strain (model 2), the OR for depression at T2 in the intervention group compared with the comparison group was 0.40 (95% CI 0.19 to 0.85, p=0.02).<br>• Stratified according to job strain at baseline the adjusted ORs for depression at T2 in the intervention group was 0.15 (95% CI 0.03 to 0.81, p=0.03) among those with job strain (model 3b).

Aikens et al. 2014[3]	**Mental Health outcomes**<br>*Stress*<br>• The intervention group rated themselves lower on perceived stress (PSS-14) and higher in resiliency (CD-RISC) than the control group at the postintervention time point (P < 0.001, P < 0.001, respectively).<br>• There was a nonsignificant, slight increase in perceived stress at six-month follow-up.<br><br>**Work Outcomes**<br>• NA<br><br>**Other outcomes**<br>• Intention to treat analysis: With regard to the Five Facets of Mindfulness Questionnaire (FFMQ) the mindfulness intervention group rated themselves significantly higher post-intervention on all facets of mindfulness (p= <0.001-0.008), with the exception of non-judgmental awareness (p=0.227) than control.<br>• Significant post-intervention increases were found in the mindfulness treatment group on all components of vigor (Shirom Vigor Scale) including physical strength (P = 0.021), cognitive liveliness (P < 0.001), and emotional energy (P = 0.027).<br>• Analysis of within-group pre- to post– effect sizes (ESs) showed improvements over the course of the trial in all measures examined.<br>• In the ITT sample, average within-group ES from baseline to postintervention ranged from d = 0.30 to 1.03 (mean = 0.67) for the intervention group and d = −0.23 to 0.25 (mean = 0.03) for wait-list controls.<br>• Six-month follow-up showed that the intervention group ES continued to increase over time, ranging from d = 0.52 to 1.08 (mean = 0.81)<br>• At follow-up, P values representing within-group changes from baseline for the intervention group were all significant (p= 0.003 to < 0.001).<br>• By follow-up, measures of mindfulness in the ITT sample had improved substantially.<br>• Improvements taken from the FFMQ ranged from an 11.2% increase in the “nonjudgmental awareness” facet from baseline, to a 25.8% increase in the “observe” facet.
Arrendondo et al. 2017[4]	**Mental Health outcomes**<br>*Perceived Stress*<br>• The intervention group rated themselves lower on perceived stress (PSS-14) and higher in resiliency (CD-RISC) than the control group at the postintervention time point (P < 0.001, P < 0.001, respectively).<br>• Significantly lower median lower scores in the interventional group compared with the control group maintained at week-20 (18.0 [17.0, 22.0] vs 30.0 [21.5, 32.0])<br><br>*Burnout (Maslach Burnout Inventory-General Survey)*<br>• Higher values observed in the interventional group compared to the control group, without statistical significance, neither at week-8 nor at week-20.

	• However, the median differences (week-8 vs. baseline) between the two groups were statistically significant in the three subscales (intervention vs. control groups) (emotional exhaustion –1.0 [–1.4, –0.4] vs. 0.0 [–0.6, 0.4], p = 0.0014; depersonalization –0.6 [–1.4, 0.2] vs. 0.2 [–0.2, 0.4], p = 0.0264), and reduced personal accomplishment (0.3 [0.0, 0.7] vs. –0.1[–0.7, 0.1], p = 0.0118). Furthermore, at week-20, these differences vs. baseline were maintained (Table S1).<br><br>**Work Outcomes**<br>• NA<br><br>**Other outcomes**<br>*Mindfulness*<br>• Significantly higher values in all subscales in the intervention vs. control groups were observed (intervention vs. control groups: 26.0 [21.0, 28.0] vs. 16.0 [12.0, 23.0] observe, 25.0 [22.0, 31.0] vs. 21.0 [17.0, 23.0] describe, 27.0 [24.0, 30.0] vs. 23.0 [19.0, 25.0] act aware, 31.0 [27.0, 36.0] vs. 25.0 [22.0, 28.0] non-judge, and 21.0 [20.0, 24.0] vs. 17.0 [13.0, 20.0] non-react), and were maintained at week-20 in all subscales ($p < 0.05$, in all cases).<br>• Significantly higher values in all subscales in the intervention vs. control groups were maintained at week-20 in all subscales ($p < 0.05$, in all cases)<br><br>*Self-compassion*<br>• Post-intervention, higher median significant values in the intervention vs. control groups were observed in all subscales of self-compassion ($p < 0.05$, in all cases), except for common humanity.<br>• At week-20, all subscales in the interventional groups showed higher significant values compared with control group ($p < 0.05$, in all cases).<br><br>*Experiences Questionnaire-Decentering*<br>• Post-intervention, a significant higher median was observed in the interventional group compared with the control group.<br>• This significant higher value was maintained at week-20.<br><br>Heart rate variability<br>• Post-intervention, the SDNN and RMSSD values at baseline and prior to the first coherent breathing (pre 1) were lower than after the first coherent breathing (post 1). At the end of the sessions, the values prior to the second coherent breathing (pre 2) were generally higher than those observed at<br>• baseline (pre 1).<br>• SDNN and RMSSD values after the second coherent breathing (post 2) showed a further increase compared to pre 2. The SDNN and RMSSD values showed a similar pattern during the eight sessions.

Das et al. 2019 [5]	**Mental Health outcomes**<br>• IG showed significantly higher adjusted percentages of participants scoring, on average, in the highest categories for the mental health SF-36 domain: mental health (p=0.027)<br><br>**Work Outcomes**<br>• IG showed significantly higher adjusted percentages of participants scoring, on average, in the highest categories for the SF-36 domain of role limitations due to physical problems (p=0.026)<br>• Proportions were similar in both groups and between-group differences were not significant for role limitations due to emotional problems.<br>**Other outcomes**<br>• Post-intervention, intervention group showed a significantly higher mean change in SF-36 vitality compared to CG (after multivariate adjustment, 12.65 vs 4.98; p=0.003)<br>• IG showed significantly higher adjusted percentages of participants scoring, on average, in the highest categories for the following SF-36 domains: General health (p=0.014), mental health (p=0.027), role limitations due to physical problems (p=0.026), and social functioning (p=0.007).<br>• Proportions were similar and between-group differences were not significant for bodily pain and role limitations due to emotional problems<br>• The adjusted change over time for Purpose in Life (PiL) was significantly higher in the IG than in the CG (P < .001)<br>• IG had statistically significant reductions in the sleep problems index I (p=0.024) and index II (p=0.021) as well as reductions in sleep disturbance (p=0.013) and higher levels of optimal sleep (p=0.004); but no significant differences were observed for other sleep measures, including sleep adequacy, quantity, somnolence, snoring, and shortness of breath.<br>• No significant differences were observed for 7 of the 8 Profile of Mood States (POMS) domains (anger, confusion, depression, tension, vigor, and summary score);<br>• IG reported a significantly greater reduction in fatigue (p=0.027).<br>• IG had a larger mean decrease in depressive symptoms (0.042), although at 6 months, there was no significant difference in the percentage of IG and CG participants classified as being at risk of clinical depression (CESD total score ≥16). The change in total activity score from baseline to 6 months did not significantly differ between IG and CG.<br>• No significant difference for BMI and cardiometabolic risk factors of HbA1c, triglycerides, LDL, and systolic blood pressure.<br>• The intervention was a significant predictor of positive change in vitality (IG = 11.67 vs CG = 7.1, p=0.038).<br>• No other measures were statistically significant.
Das et al. 2020 [6]	**Mental Health Outcomes**<br><br>• IG showed significantly higher adjusted percentages of participants scoring, on average, in the highest categories for the mental health SF-36 domain: mental health (p<0.001 at 6; 12 and 18 months follow-up)

	• Compared to baseline, mean depression scores (CESD total scores) were significantly lower at months 6 and 12 (P <.0001 for both time points) and at month 18 (P=0.0003). There was also a modest reduction in the prevalence of depression at months 6, 12, and 18 compared to baseline, which was statistically significant at months 6 and 12 (P < .05) after adjustment for multiple comparisons, but not at month 18.<br>**Work Outcomes**<br>• NA<br><br>**Other outcomes**<br>• At 18-month follow-up, there were sustained improvements vitality and general health domains of SF-36 and PiL (P < .001 for all measures). Sleep, mood, vigor, physical activity, and blood pressure were also improved at 18 months (P < .05 for all measures).<br>• Participants reported less sleep disturbance and better sleep quality at months 6 and 12 (P< .05); better sleep adequacy at months 6, 12, and 18 months (P < .05) compared to baseline; higher mean prevalence of optimal sleep at month 6 compared to baseline (P =0.003), but this difference was not sustained at month 12 or 18.<br>• No statistically significant difference was observed for shortness of breath at months 6, 12, or 18 compared to baseline.<br>• Mean walking MET minutes were not significantly different from baseline for months 6 and 12 but were significantly different for month 18 (P= 0.018. Participants showed a statistically significant increase in total physical activity score (IPAQ score) at months 6, 12, and 18 compared to baseline (P =<br>0.0035, .0001, <.0001, respectively<br>• There were no statistically significant differences from baseline at months 6, 12, and 18 for body weight, BMI, and body fat. Waist-to-hip ratio was significant at months 12 and 18 compared to baseline (P= 0.031). Mean hip circumference was statistically lower at months 6, 12, and 18 compared<br>• to baseline (P < .0001 for all time points). Waist circumference, on average, was lower at months 6, 12, and 18 and achieved statistical significance at months 12 (P <.001) and 18 (P = 0.041).
Dollard et al. 2014[22]	**Mental Health Outcomes**<br>• In terms of effects on stress outcomes morale improved, and sickness absence duration decreased in the PAR intervention group compared with the control group; work stress did not change across the workgroups.<br><br>**Work Outcomes**<br>• organizational and job design factors improved in the intervention workgroups relative to the control workgroups.<br><br>**Other outcomes**<br>• Participants very positively rated the participative process of the intervention workshops.
Formanoy et al. 2016[8]	**Mental Health Outcomes**<br>• NA

	**Work Outcomes**<br>• office workers in the social environmental intervention group showed a mean improvement in NFR of 3.82, and those who did not receive the social environmental intervention showed a mean improvement of 1.17 (Table 2); the difference in means was 2.65 (SD = 23.63; effect size d = 0.11), and the main effect of the social environmental intervention was not significant (p >0.05; indicated by an independent t-test).<br>• The office workers in the physical environmental intervention group showed a mean improvement in NFR of 4.59, and those who did not receive the physical environmental intervention showed a mean increase of 0.85 (Table 2); the difference in means was 3.75 (SD = 23.60; effect size d = 0.16), and the main effect of the physical environmental intervention was not significant (p > 0.05; indicated by<br>• The results indicated that younger office workers (≤46.5 years) who received the social environmental intervention had a better outcome in NFR than younger office workers who did not receive the social environmental intervention (Leaf 1 in Fig. 1; difference in means = 10.52; 95 % CI: 4.12, 16.92); bias-corrected effect size d = 0.32,Table 3).<br>• Furthermore, the results showed that older office workers (> 46.5 years) who received the social environmental intervention had a worse outcome than older office workers who did not receive the social environmental intervention (Leaf 2 in Fig. 1; difference in means = -10.65; 95 % CI: −19.35, −1.96); bias-corrected effect size d = -0.22, Table 3).<br>• The results indicated<br>• that office workers who worked fewer hours overtime (≤ 2.25) had a better outcome with the physical environmental intervention than without the physical environmental intervention (Leaf 1 in Fig. 2; difference in means = 7.40; 95 % CI: 0.99, 13.81); bias corrected effect size d=0.13).<br>• Furthermore, the results showed that office workers who worked more hours overtime (> 2.25) had a worse outcome with the physical environmental intervention than without, but this effect was not significant (Leaf 2 in Fig. 2; difference in means = −6.95; 95 % CI: −16.26, 2.36); bias-corrected effect size d = −0.08, Table 3).<br><br>**Other outcomes**<br>• NA
Grégoire, and Lachance. 2015[9]	**Mental Health Outcomes**<br>*Psychological distress*<br>• Simple effect analyses revealed no differences between the groups at t1 regarding negative affect and fatigue. At t2, the employees in group 1 (intervention arm) experienced significantly less negative affect and fatigue than employees in group 2 (control arm), while at t3, these differences were no longer significant.<br><br>**Work Outcomes**<br>• NA<br><br>**Other outcomes**<br>*Mindfulness*

	• Employees in group 2 (control) were significantly more mindful than those in group 1 (intervention) at baseline. Regarding the main effect for time, Bonferroni pairwise comparisons (.01 level) revealed that the mean mindfulness scores were higher at t3 than those at t1, regardless of the group.<br>• As employees developed their mindfulness skills, their psychological distress tends to diminish (There was a negative correlation between the mindfulness gain scores and the gain scores for all the indicators of psychological distress (stress: r=−.50, p<.001; anxiety/depression: r=−.56, p<.001; fatigue: r=−.62, p<.001; negative affect:r=−.46, p<.001) with the strength of the association varying from moderate to strong)
Hasson et al. 2010[10]	**Mental Health Outcomes**<br>*Stress*<br>• At the midpoint of the study, there was a trend (P = 0.10) toward the low-use of a worksite web-based program for stress management group reporting higher levels of stress (mean 54.8) compared with the high-use group (mean 48.7). By the end of the program, this trend had firmed to a significantly (P = 0.05) higher reporting of stress in the low-use group (mean 53.0) compared with the high-use group (mean 45.1).<br><br>**Work Outcomes**<br>• NA<br><br>**Other outcomes**<br>• **NA**
Kojima et al. 2010[11]	**Mental Health Outcomes**<br>*Depression*<br>• CES-D score decreased by 2.21 points in the intervention group but increased by 0.12 points in the control group, showing a significant between-group difference of –2.33 points (95% confidence interval [CI]: –3.89 to –0.77; p<0.001)<br>*Understanding stress control skills*<br>• Understanding stress control skills increased by 1.06 points in the intervention group and by 0.04 points in the control group, showing a significant between-group difference of 1.02 (95% CI: 0.81 to 1.23; p<0.001).<br>*Applying stress control skills*<br>• Scores for will to apply these stress control skills ("I am trying to expand the repertoire of my ways of thinking by considering things from different points of view") increased by 0.42 points in the intervention group and by 0.07 points in the control group, showing a significant between-group difference of 0.35 points (95% CI: 0.14 to 0.56; p<0.01).<br><br>**Work Outcomes**<br>• NA<br><br>**Other outcomes**<br>*Self-esteem*

	• Self-esteem score increased by 1.73 points in the intervention group and by 0.76 points in the control group, showing a non-significant between-group difference of 0.97 points (95% CI: –2.17 to 0.23; p=0.11)
Lloyd et al. 2017[12]	**Mental Health Outcomes**<br>No significant changes in these variables were observed in the control group.<br><br>*Psychological Strain*<br>• there was a significant group by time interaction for psychological strain. Within-groups simple contrasts indicated that in the SMT group there was a significant decrease in psychological strain between Time 1 and Time 2 ($F(1, 67) = 4.60$, $p < .05$, $\eta^2 = .06$), and between Time 1 and Time 3 ($F(1, 67) = 4.42$, $p < .05$, $\eta^2 = .06$), while no significant changes in psychological strain were observed in the control group. Between-groups simple effects contrasts, with Time 1 psychological strain scores entered as a covariate, showed that psychological strain was significantly lower in the SMT group at Time 2 ($F(1, 150) = 8.29$, $p < .01$, $\eta^2 = .05$), and at Time 3 ($F(1, 150) = 6.68$, $p < .01$, $\eta^2 = .04$)<br><br>*Emotional Exhaustion*<br>• As can be seen in Table 2, there was a significant main effect for time, and a significant group by time interaction, for emotional exhaustion. Within-groups simple contrasts indicated that in the SMT group there was a significant decrease in emotional exhaustion between Time 1 and Time 2 ($F(1, 67)= 6.89$, $p < .01$, $\eta^2 = .09$), and between Time 1 and Time 3 ($F(1, 67) = 18.88$, $p < .001$, $\eta^2 = .22$), while no significant changes in emotional exhaustion were observed in the control group. Between groups simple effects contrasts, with Time 1 emotional exhaustion scores entered as a covariate, showed that emotional exhaustion was significantly lower in the SMT group at Time 3 ($F(1, 150) = 5.45$, $p < .05$, $\eta^2 = .04$), but not at Time 2<br><br>*Depersonalization*<br>• As can be seen in Table 2, there was a significant group by time interaction for depersonalization. Within-groups simple contrasts indicated that in the SMT group there was a significant decrease in depersonalization between Time 1 and Time 3 ($F(1, 67) = 4.43$, $p < .05$, $\eta^2 = .06$), but not between Time 1 and Time 2, while no significant changes in depersonalization were observed in the control group. Between-groups simple effects contrasts, with age, time in line of work and Time 1 depersonalization scores Work-related self-efficacy as a moderator of SMT entered as covariates, showed that depersonalization was significantly lower in the ACT group at Time 3 ($F(1, 148) = 4.29$, $p < .05$, $\eta^2 = .03$), but not at Time 2.<br><br>**Work Outcomes**<br>• NA<br><br>**Other outcomes**<br>• NA

Michishita et al. 2017[13]	**Mental Health Outcomes**<br>*Mood states*<br>• After 10 weeks, the items of "fatigue-inertia" decreased and " vigor-activity " and " friendliness " in POMS 2 increased in the intervention group (p<0.05). A significant interaction effect for group × time was seen in the items of " fatigue-inertia " , " vigor-activity " and " friendliness " in POMS 2 between the two groups (p<0.05).<br>*Job-related stress*<br>• The BJSQ items of "interpersonal stress" decreased and "vigor," "support from superiors, colleagues and family/friends," and "satisfaction with job/daily life" increased in the intervention group (p<0.05). A significant interaction effect for group × time was seen in the BJSQ items of "interpersonal stress," "vigor," "support from superiors, colleagues and family/friends," and "satisfaction with job/daily life" between the two groups (p<0.05).<br><br>**Work Outcomes**<br>*Work ability*<br>• No significant difference in work ability<br><br>**Other Outcomes**<br>• The BJSQ items of "support from superiors, colleagues and family/friends," and "satisfaction with job/daily life" increased in the intervention group (p<0.05). A significant interaction effect for group × time was seen in the BJSQ items of "support from superiors, colleagues and family/friends," and "satisfaction with job/daily life" between the two groups (p<0.05).<br>• In the intervention group, the number of exercise participation was positively correlated with the change in "vigor-activity" in POMS 2 (r=0.467, p=0.011).
Mills 2007[14]	**Mental Health Outcomes**<br>• NA<br>**Work Outcomes**<br>*Work Performance*<br>• The average score on the work performance scale increased significantly in the intervention group by 0.61 points (from a baseline mean of 7.6).<br>• No significant changes occurred in any of the comparable outcomes in the weighted control group.<br>• Improvements in work performance were significantly greater in the intervention group than the control group in regression analysis: a greater average reduction of close to one point on the 0 to 10 scale of work performance (0.79). *Sickness absence*<br>• No significant changes occurred in any of the comparable outcomes in the weighted control group.<br>• Improvements in sickness absence were significantly greater in the intervention group than the control group in regression analysis: a greater average reduction of more than one-third of a monthly absenteeism day (0.36). In the case of absenteeism, the significant effect was due more to an increase in the control group than to a decrease in the intervention group.

	**Other Outcomes**<br>• The average number of health risk factors assessed in the HRA decreased significantly in the weighted intervention group between baseline and the 12-month follow-up, with a mean decrease of 20.48 health risk factors.<br>• Improvements in health risk factors were significantly greater in the intervention group than the control group in regression analysis: a greater average reduction of nearly one-half a health risk factor (0.45
Munz 2001[23]	**Mental Health Outcomes**<br>*Perceived Stress*<br>• The self-management training group showed significantly less perceived stress than the control group on the PSS post-measure [F (1, 74) = 11.72, p < .05].<br>*Depression*<br>• the training group showed less depression on the CES-D post-measure than the control group [F (1, 75) =8.03, p < .05].<br><br>**Work Outcomes**<br>• The training group did report significantly higher scores than the control group on the WAS subscale of Job Independence [F (1,73) =8.53, p < .05].<br>*Work Group Productivity*<br>• the treatment work units showed an average of 23% improvement in sales (revenue per order) compared to the control work unit's 17% increase.<br>*Absenteeism*<br>• the treatment group's work units showed an average of 24% reduction in absenteeism as compared to the control work unit's 7% drop.<br><br>**Other Outcomes**<br>• For affect, the training group showed significantly less negative affect (negative arousal) than the control on the PANAS post-measure [F (1,74) = 13.09, p< .05)]. Also, the training group reported significantly less low negative affect (relaxation) than the control group [F (1,74) = 8.48, p < .05]. For positive affect, the training group had significantly higher scores (positive energy) than the control group [F (1,74) = 4.16, p < .05]. For low positive affect, the training group reported significantly less tiredness than the control group [F(1, 67) = 10.56, p < .05].
Saavedra 2021[16]	**Mental Health**<br>• There was an improvement in mental health (4.760 ≤ F ≤ 8.087, 0.008 ≤ p ≤ 0.037)<br>• Both the circuit training (CT) and brisk walking (BW) groups showed reduced levels of depression, anxiety, and stress, and this reduction was also present in the control © group.<br>**Work Outcomes**<br>• NA<br>**Other Outcomes**<br>• Both exercise programs (CT and BW) maintained the body weight and body mass index while reducing body fat mass (a group × time interaction; 4.864 ≤ F ≤ 6.524, 0.001 ≤ p ≤ 0.015), although the CT intervention also showed relevant (inter-group) reductions in the waist-hip

	ratio (F = 11.311, p =0.007) and increased skeletal muscle mass (F = 15.062, p = 0.003). Both exercise programs (CT and BW) improved the cardiorespiratory fitness test scores (a group × time interaction; F = 18.054, p < 0.001). There were no changes in the lipid profile or blood pressure after the interventions
Saelid 2016[17]	**Mental Health**<br>*Depression*<br>• The Coping With Strain (CWS) course significantly reduces symptoms of depression among those who participate, and the effects are maintained four years after the intervention. The effects of CWS in reducing depressive symptoms were estimated from three models, including calculation of effect sizes.<br>• Across groups, participants on average experienced a reduction of 3.46 points on the Beck Depression Inventory (BDI) scale in the 8 weeks the course lasted (p < 0.001).<br>• While participating in the CWS course, effect sizes in IG1 (M1–M2) and II (M3–M4) showed some effect, respectively 0.51 and 0.40. In IG2 there was a low effect (0.15) while the participants were waiting for the course to start (M1–M3). The effect size revealed some effect (0.35) between IG1 at M2 and the delayed intervention (IG2) at M3. The effect size showed moderate or high effect on all follow-ups in both intervention groups after participating in the CWS course.<br>**Work Outcomes**<br>• NA<br>**Other Outcomes**
Smith 2008[24]	**Mental Health**<br>*Anxiety*<br>• Participants in the music relaxation intervention revealed a significant reduction in state anxiety scores post assessment compared to pre assessment t(df0=16.8(30), p=<0.01. There was a 21 point reduction (CI=18.5=23.5) in state anxiety level post intervention. There was no significant change in pre and post scores for the discussion group.<br><br>**Work Outcomes**<br>• NA<br><br>**Other Outcomes**<br>*Relaxation & Pleasantness*<br>• Participants in the music relaxation intervention indicated increased relaxation t(df)=-16.2, p=<0.01) and pleasantness (t(df)=-20.27(39), p=<0.01); as well as decreased tension t(df)=12.0(39), p=<0.01) from pre to post intervention
Takao 2006[19]	**Mental Health**<br>*Psychological distress*

	• The intervention effects (time x group) were not significant for psychological distress among both male (p=0.456) and female (p=0.714) subordinates<br>• Young male subordinates engaged in white-collar occupations showed significant intervention effects for psychological distress (p=0.012)<br><br>**Work Outcomes**<br>*Job Performance*<br>• The intervention effects (time x group) were not significant for job performance among both male (p= 0.252) and female (p= 0.106) subordinates<br>• Young male subordinates engaged in white-collar occupations showed significant intervention effects for job performance (p=0.029)<br><br>**Other Outcomes**<br>• NA
Workman 2004[25]	**Mental Health**<br>• NA<br><br>**Work Outcomes**<br>*Job Satisfaction*<br>• Employees receiving the alignment job design (AJD) intervention experienced increased job satisfaction (compared to the control group) (d=0.98, p<0.01).,<br>• Employees in the high-involvement work processes (HIWP) intervention experienced significantly improved job satisfaction (d=1.54 (p<0.001) when compared to the control group on these same measures.<br>• The autonomous work teams' intervention did not improve employee job satisfaction (relative to the control group).<br>Organizational Commitment<br>• Employees receiving the alignment job design (AJD) intervention showed no difference in commitment (compared to the control group<br>• Employees in the high-involvement work processes (HIWP) intervention experienced a significant d score (d=0.75, p<0.01) for organizational commitment when compared to the control group on these same measures.<br>• The autonomous work teams' intervention did not improve organizational commitment (relative to the control group).<br><br>**Other Outcomes**<br>• NA

Table S4. PRISMA-ScR Checklist

Preferred Reporting Items for Systematic reviews and Meta-Analyses extension for Scoping Reviews (PRISMA-ScR) Checklist

SECTION	ITEM	PRISMA-ScR CHECKLIST ITEM	REPORTED ON PAGE #
TITLE			
Title	1	Identify the report as a scoping review.	1
ABSTRACT			
Structured summary	2	Provide a structured summary that includes (as applicable): background, objectives, eligibility criteria, sources of evidence, charting methods, results, and conclusions that relate to the review questions and objectives.	2
INTRODUCTION			
Rationale	3	Describe the rationale for the review in the context of what is already known. Explain why the review questions/objectives lend themselves to a scoping review approach.	6
Objectives	4	Provide an explicit statement of the questions and objectives being addressed with reference to their key elements (e.g., population or participants, concepts, and context) or other relevant key elements used to conceptualize the review questions and/or objectives.	6
METHODS			
Protocol and registration	5	Indicate whether a review protocol exists; state if and where it can be accessed (e.g., a Web address); and if available, provide registration information, including the registration number.	NA
Eligibility criteria	6	Specify characteristics of the sources of evidence used as eligibility criteria (e.g., years considered, language, and publication status), and provide a rationale.	7
Information sources*	7	Describe all information sources in the search (e.g., databases with dates of coverage and contact with authors to identify additional sources), as well as the date the most recent search was executed.	8
Search	8	Present the full electronic search strategy for at least 1 database, including any limits used, such that it could be repeated.	Search Strategy section in supplementary material
Selection of sources of evidence†	9	State the process for selecting sources of evidence (i.e., screening and eligibility) included in the scoping review.	8
Data charting process‡	10	Describe the methods of charting data from the included sources of evidence (e.g., calibrated forms or forms that have been tested by the team before their use, and whether data charting was done independently or in duplicate) and any processes for obtaining and confirming data from investigators.	9
Data items	11	List and define all variables for which data were sought and any assumptions and simplifications made.	9
Critical appraisal of individual	12	If done, provide a rationale for conducting a critical appraisal of included sources of evidence; describe	NA

St. Michael's
Inspired Care.
Inspiring Science.

SECTION	ITEM	PRISMA-ScR CHECKLIST ITEM	REPORTED ON PAGE #
sources of evidence§		the methods used and how this information was used in any data synthesis (if appropriate).	
Synthesis of results	13	Describe the methods of handling and summarizing the data that were charted.	9-10
**RESULTS**			
Selection of sources of evidence	14	Give numbers of sources of evidence screened, assessed for eligibility, and included in the review, with reasons for exclusions at each stage, ideally using a flow diagram.	Figure 1
Characteristics of sources of evidence	15	For each source of evidence, present characteristics for which data were charted and provide the citations.	Table 1
Critical appraisal within sources of evidence	16	If done, present data on critical appraisal of included sources of evidence (see item 12).	NA
Results of individual sources of evidence	17	For each included source of evidence, present the relevant data that were charted that relate to the review questions and objectives.	Table 1
Synthesis of results	18	Summarize and/or present the charting results as they relate to the review questions and objectives.	Tables 1-3
**DISCUSSION**			
Summary of evidence	19	Summarize the main results (including an overview of concepts, themes, and types of evidence available), link to the review questions and objectives, and consider the relevance to key groups.	17
Limitations	20	Discuss the limitations of the scoping review process.	19
Conclusions	21	Provide a general interpretation of the results with respect to the review questions and objectives, as well as potential implications and/or next steps.	20
**FUNDING**			
Funding	22	Describe sources of funding for the included sources of evidence, as well as sources of funding for the scoping review. Describe the role of the funders of the scoping review.	20

JBI = Joanna Briggs Institute; PRISMA-ScR = Preferred Reporting Items for Systematic reviews and Meta-Analyses extension for Scoping Reviews.

* Where *sources of evidence* (see second footnote) are compiled from, such as bibliographic databases, social media platforms, and Web sites.

† A more inclusive/heterogeneous term used to account for the different types of evidence or data sources (e.g., quantitative and/or qualitative research, expert opinion, and policy documents) that may be eligible in a scoping review as opposed to only studies. This is not to be confused with *information sources* (see first footnote).

‡ The frameworks by Arksey and O'Malley (6) and Levac and colleagues (7) and the JBI guidance (4, 5) refer to the process of data extraction in a scoping review as data charting.

§ The process of systematically examining research evidence to assess its validity, results, and relevance before using it to inform a decision. This term is used for items 12 and 19 instead of "risk of bias" (which is more applicable to systematic reviews of interventions) to include and acknowledge the various sources of evidence that may be used in a scoping review (e.g., quantitative and/or qualitative research, expert opinion, and policy document).

From: Tricco AC, Lillie E, Zarin W, O'Brien KK, Colquhoun H, Levac D, et al. PRISMA Extension for Scoping Reviews (PRISMAScR): Checklist and Explanation. Ann Intern Med. 2018;169:467–473. doi: 10.7326/M18-0850.

REFERENCES

1. Agarwal U, Mishra S, Xu J, et al. A Multicenter Randomized Controlled Trial of a Nutrition Intervention Program in a Multiethnic Adult Population in the Corporate Setting Reduces Depression and Anxiety and Improves Quality of Life: The GEICO Study. *Am J Health Promot* 2015; 29: 245-254. DOI: 10.4278/ajhp.130218-QUAN-72.
2. Ahola K, Vuori J, Toppinen-Tanner S, et al. Resource-enhancing group intervention against depression at workplace: who benefits? A randomised controlled study with a 7-month follow-up. *Occup Environ Med* 2012; 69: 870-876. 2012/06/22. DOI: 10.1136/oemed-2011-100450.
3. Aikens KA, Astin J, Pelletier KR, et al. Mindfulness goes to work: impact of an online workplace intervention. *J Occup Environ Med* 2014; 56: 721-731. 2014/07/06. DOI: 10.1097/jom.0000000000000209.
4. Arredondo M, Sabaté M, Valveny N, et al. A mindfulness training program based on brief practices (M-PBI) to reduce stress in the workplace: a randomised controlled pilot study. *Int J Occup Environ Health* 2017; 23: 40-51. 2017/10/31. DOI: 10.1080/10773525.2017.1386607.
5. Das SK, Mason ST, Vail TA, et al. Effectiveness of an Energy Management Training Course on Employee Well-Being: A Randomized Controlled Trial. *Am J Health Promot* 2019; 33: 118-130. 2018/05/29. DOI: 10.1177/0890117118776875.
6. Das SK, Mason ST, Vail TA, et al. Sustained Long-Term Effectiveness of an Energy Management Training Course on Employee Vitality and Purpose in Life. *Am J Health Promot* 2020; 34: 177-188. 2019/11/09. DOI: 10.1177/0890117119883585.
7. Dollard MF and Gordon JA. Evaluation of a participatory risk management work stress intervention. *Int J Stress Manag* 2014; 21: 27-42. DOI: 10.1037/a0035795.
8. Formanoy MA, Dusseldorp E, Coffeng JK, et al. Physical activity and relaxation in the work setting to reduce the need for recovery: what works for whom? *BMC Public Health* 2016; 16: 866. 2016/08/26. DOI: 10.1186/s12889-016-3457-3.
9. Grégoire S and Lachance L. Evaluation of a Brief Mindfulness-Based Intervention to Reduce Psychological Distress in the Workplace. *Mindfulness* 2015; 6: 836-847. DOI: 10.1007/s12671-014-0328-9.
10. Hasson H, Brown C and Hasson D. Factors associated with high use of a workplace web-based stress management program in a randomized controlled intervention study. *Health Educ Res* 2010; 25: 596-607. 2010/02/13. DOI: 10.1093/her/cyq005.
11. Kojima R, Fujisawa D, Tajima M, et al. Efficacy of cognitive behavioral therapy training using brief e-mail sessions in the workplace: a controlled clinical trial. *Ind Health* 2010; 48: 495-502. 2010/08/20. DOI: 10.2486/indhealth.ms1135.
12. Lloyd J, Bond FW and Flaxman PE. Work-related self-efficacy as a moderator of the impact of a worksite stress management training intervention: Intrinsic work motivation as a higher order condition of effect. *J Occup Health Psychol* 2017; 22: 115-127. 2016/04/08. DOI: 10.1037/ocp0000026.
13. Michishita R, Jiang Y, Ariyoshi D, et al. The practice of active rest by workplace units improves personal relationships, mental health, and physical activity among workers. *J Occup Health* 2017; 59: 122-130. 2016/12/17. DOI: 10.1539/joh.16-0182-OA.
14. Mills PR, Kessler RC, Cooper J, et al. Impact of a health promotion program on employee health risks and work productivity. *Am J Health Promot* 2007; 22: 45-53. 2007/09/27. DOI: 10.4278/0890-1171-22.1.45.
15. Munz DC, Kohler JM and Greenberg CI. Effectiveness of a Comprehensive Worksite Stress Management Program: Combining Organizational and Individual Interventions. *Int J Stress Manag* 2001; 8: 49-62.
16. Saavedra JM, Kristjánsdóttir H, Gunnarsson SB, et al. Effects of 2 physical exercise programs (circuit training and brisk walk) carried out during working hours on multidimensional components of workers' health: a pilot study. *Int J Occup Med Environ Health* 2021; 34: 39-51. 2020/11/11. DOI: 10.13075/ijomeh.1896.01647.

17. Saelid GA, Czajkowski NO, Holte A, et al. Coping With Strain (CWS) course - its effects on depressive symptoms: A four-year longitudinal randomized controlled trial. *Scand J Psychol* 2016; 57: 321-327. 2016/04/29. DOI: 10.1111/sjop.12289.
18. Smith M. The effects of a single music relaxation session on state anxiety levels of adults in a workplace environment. *AJMT* 2008; 19: 45-66.
19. Takao S, Tsutsumi A, Nishiuchi K, et al. Effects of the job stress education for supervisors on psychological distress and job performance among their immediate subordinates: a supervisor-based randomized controlled trial. *J Occup Health* 2006; 48: 494-503. 2006/12/21. DOI: 10.1539/joh.48.494.
20. Workman M and Bommer W. Redesigning computer call center work: a longitudinal field experiment. *J Organ Behav* 2004; 25: 317-337. DOI: https://doi.org/10.1002/job.247.
21. Agarwal U, Mishra S, Xu J, et al. A Multicenter Randomized Controlled Trial of a Nutrition Intervention Program in a Multiethnic Adult Population in the Corporate Setting Reduces Depression and Anxiety and Improves Quality of Life: The GEICO Study. *American Journal of Health Promotion* 2015; 29: 245-254. DOI: 10.4278/ajhp.130218-QUAN-72.
22. Dollard MF and Gordon JA. Evaluation of a participatory risk management work stress intervention. *International Journal of Stress Management* 2014; 21: 27-42. DOI: 10.1037/a0035795.
23. Munz DC, Kohler JM and Greenberg CI. Effectiveness of a Comprehensive Worksite Stress Management Program: Combining Organizational and Individual Interventions. *International Journal of Stress Management* 2001; 8: 49-62.
24. Smith M. The effects of a single music relaxation session on state anxiety levels of adults in a workplace environment. *Australian Journal of Music Therapy* 2008; 19: 45-66.
25. Workman M and Bommer W. Redesigning computer call center work: a longitudinal field experiment. *Journal of Organizational Behavior* 2004; 25: 317-337. DOI: https://doi.org/10.1002/job.247.
